# A Uniform and Isotropic Cytoskeletal Tiling Fills Dendritic Spines

**DOI:** 10.1523/ENEURO.0342-22.2022

**Published:** 2022-10-26

**Authors:** Florian Eberhardt, Eric A. Bushong, Sébastien Phan, Steven Peltier, Pablo Monteagudo-Mesas, Tino Weinkauf, Andreas V. M. Herz, Martin Stemmler, Mark Ellisman

**Affiliations:** 1 Faculty of Biology, Ludwig-Maximilians-Universität and Bernstein Center for Computational Neuroscience Munich, Munich, Planegg-Martinsried D-82152, Germany; 2 Department of Neurosciences and National Center for Microscopy and Imaging Research, Center for Research in Biological Systems, University of California, San Diego, La Jolla, 92093 CA; 3 Department of Neurosciences, University of California, San Diego, La Jolla, 92093 CA; 4 School of Electrical Engineering and Computer Science, KTH Royal Institute of Technology, Stockholm, 100 44 Sweden

**Keywords:** actin cytoskeleton, cerebellar Purkinje cell, dendritic spines *in situ*, EM tomography, hippocampal pyramidal cell, image segmentation

## Abstract

Dendritic spines are submicron, subcellular compartments whose shape is defined by actin filaments and associated proteins. Accurately mapping the cytoskeleton is a challenge, given the small size of its components. It remains unclear whether the actin-associated structures analyzed in dendritic spines of neurons *in vitro* apply to dendritic spines of intact, mature neurons *in situ.* Here, we combined advanced preparative methods with multitilt serial section electron microscopy (EM) tomography and computational analysis to reveal the full three-dimensional (3D) internal architecture of spines in the intact brains of male mice at nanometer resolution. We compared hippocampal (CA1) pyramidal cells and cerebellar Purkinje cells in terms of the length distribution and connectivity of filaments, their branching-angles and absolute orientations, and the elementary loops formed by the network. Despite differences in shape and size across spines and between spine heads and necks, the internal organization was remarkably similar in both neuron types and largely homogeneous throughout the spine volume. In the tortuous mesh of highly branched and interconnected filaments, branches exhibited no preferred orientation except in the immediate vicinity of the cell membrane. We found that new filaments preferentially split off from the convex side of a bending filament, consistent with the behavior of Arp2/3-mediated branching of actin under mechanical deformation. Based on the quantitative analysis, the spine cytoskeleton is likely subject to considerable mechanical force *in situ*.

## Significance Statement

The actin-cytoskeleton lends shape to a spine and compartmentalizes its intracellular space. By using state-of-the-art three-dimensional (3D) electron microscopy (EM), we reconstructed the 3D internal architecture of dendritic spines *in situ,* revealing that the cytoskeleton is subject to higher mechanical stress than what *in vitro* studies suggest. In fact, a detailed quantitative analysis shows that putative actin forms part of a uniform mesh-gel throughout the spine neck and spine head *in situ*. The high-resolution imaging and reconstruction of the internal structure reveals the likely constraints on the flow of molecules within dendritic spines, with consequences for electrochemical signaling, synaptic plasticity, learning, and memory.

## Introduction

Dendritic spines are small membrane protrusions whose shapes are supported by a highly dynamic cytoskeleton that consists primarily of filamentous actin (Fifková and Delay, 1982; Matus et al., 1982; Capani et al., 2001a,b,c, 2005, 2008; Ouyang et al., 2005). Spine shape and size correlate with postsynaptic current flow into the dendritic tree. Not only does the spine’s gross morphology, the size and shape of the spine head, the length and diameter of the spine neck, influence the flow of electric currents and active transport processes of molecules within the spine, but so should the fine structure of the spine’s cytoskeleton. Yet little is known about the three-dimensional (3D) architecture at high resolution, as most of what we know is either derived from electron microscopy (EM) studies on cultured neurons, from ultrastructurally compromised immunolocalization studies or from resolution-limited light-microscopic analyses.

The actin cytoskeleton within a spine head branches frequently (Korobova and Svitkina, 2010; Bellot et al., 2014; Chazeau and Giannone, 2016; Lei et al., 2016; Basu and Lamprecht, 2018). During the formation and continual reorganization of the actin mesh that supports protrusions and invaginations of the cell membrane (Simon et al., 2019), new filaments branch off at 70° angles, as mediated by the Arp2/3 complex (Mullins et al., 1998; Blanchoin et al., 2000) that is found at high concentrations in spine heads (Hotulainen et al., 2009). In contrast, CaMKII, myosin, and other molecules cause actin to form linear bundles (Okamoto et al., 2007; Kasai et al., 2021), a process that is dynamically regulated (Khan et al., 2019). A common hypothesis is that the spine neck in mature spines might maintain a system of unbranched actin filaments aligned with the neck’s principal axis (Rochefort and Konnerth, 2012; Bellot et al., 2014; Konietzny et al., 2017; Obashi et al., 2021); however, Korobova and Svitkina (2010) find few examples of longer longitudinal filaments in dendritic spine necks. High-resolution reconstruction could quantitatively assess whether different pools of actin in spines (Honkura et al., 2008) lead to measurable differences in the cytoskeleton (comprising actin, actin-binding and actin-associated proteins) across different spine domains.

Recent advances in electron microscopic tomography and automated segmentation techniques provide an opportunity to study the 3D internal architecture at nanometer resolution. In this first detailed electron tomographic analysis of the three-dimensional (3D) fine structure of dendritic spines based on *in situ* preparations, we imaged two major classes of dendritic spines, spines on hippocampal (CA1) pyramidal cells and spines on cerebellar Purkinje cells. The spine ultrastructure we imaged contains not only the bifurcating filaments of actin, but also cross-linking proteins. Together, actin and cross-links define the mechanical and electrostatic properties of a network of filaments. We studied the properties of individual branches in this network (branch length, branch tortuosity, branch orientation with respect to the membrane) as well as their contributions to the overall shape of the mesh (connectivity, branching angles, elementary loops formed by the filaments). We quantified a surprising degree of uniformity and isotropy of the cytoskeleton; despite overall size differences, the architecture of hippocampal and cerebellar spines is quite similar. Moreover, the filamentous scaffolds in the spine neck and spine head are quantitatively similar, which confirms the report by Korobova and Svitkina (2010) and stands in contrast to other results from spines in cultured neurons.

Previous modeling focused on a spine’s gross structure (Byrne et al., 2011). Our findings provide spatially resolved structural information for refined functional, computational, and theoretical models of excitatory synaptic transmission that will capture the detailed spatiotemporal dynamics and computations within a spine.

## Materials and Methods

### Animals and tissue preparation

Tissue was collected from C57BL/6NHsd two male mice (Envigo) that were treated in accordance with a protocol approved by the Institutional Animal Care and Use Committee at the University of California, San Diego. Animals were one month (cerebellum) and two months (hippocampus) old. They were anesthetized with ketamine/xylazine and then transcardially perfused with Ringer’s solution followed by fixative containing 2.5% glutaraldehyde, 2% formaldehyde, 2 mm CaCl_2_, and 0.15 m cacodylate buffer. The fixation was started at 37°C and the fixative was cooled to ice temperature during the 15-min perfusion. The brain was removed and postfixed in the same fixative for 1 h. The brains were sectioned using a Vibratome (Ted Pella) into 100-μm slices, which were briefly stored in 0.15 m Na cacodylate buffer with 2 mm CaCl_2_ before high pressure freezing.

### High-pressure freezing and freeze substitution

A tissue punch was used to remove a portion of either cerebellum or hippocampus from tissue slices. The punch was placed into a 100-μm-deep membrane carrier and surrounded with 20% BSA in 0.15 m Na cacodylate buffer. The specimens were then frozen with either a Leica EM PACT2 (cerebellum) or a Baltec HP10 (hippocampus). The specimens were stored in liquid nitrogen before freeze substitution. Freeze substitution was conducted in extra dry acetone (Acros) as follows: 0.1% tannic acid at −90°C for 24 h, wash 3 × 20 min in acetone at −90°C, transferred to 2% osmium tetroxide (OsO_4_)/0.1% uranyl acetate (UA) and kept at −90°C for 48 h, warmed to −60°C for 15 h, kept at −60°C for 10 h, warmed to 0°C for 16 h. The specimens were then washed with ice-cold acetone and allowed to come to room temperature and washed twice more with acetone. The specimens were infiltrated with 1:3 Durcupan ACM resin:acetone for several hours, 1:1 Durcupan:acetone for 24 h, 3:1 Durcupan:acetone for several hours, 100% Durcupan:acetone overnight, and then fresh Durcupan for several hours. The 100% Durcupan steps were done under vacuum. The specimens were then placed in Durcupan in 60°C oven for 48 h.

### EM specimen preparation

The epoxy blocks were cut with a Leica UCT ultramicrotome into 300-nm semi-thick sections. There was no need for to perform on grid heavy metal staining as the en bloc staining was sufficient. Ribbons were collected on previously glow discharged slot grids with a 50-nm-thick support film (Luxel Corp). The grids were glow discharged again for 10 s on both sides and then coated with 10 nm colloidal gold diluted 1:2 with 0.05 m bovine serum albumin solution (Ted Pella).

### Electron microscopic tomography

For imaging, the plastic embedded specimen sections were inserted in an intermediate-voltage transmission electron microscope operating at 300 kV (FEI Titan high base) with a 4k × 4k CCD detector (Gatan Ultrascan). Portions of brain tissues containing dendritic spines were digitally reconstituted following the multitilt electron tomography protocol described by Phan et al. (2017). Specifically, tomograms were generated from four tilt series, with the angle characterizing the azimuthal sample orientation taken, respectively, at 0°, 90°, 45°, and 135°. For each series, the grid supporting the specimen sections was tilted around the holder axis, usually from −60° to +60° with a 1° increment. With a pixel-size of ∼0.4 nm, a typical tomogram would span a range of 1.5 μm × 1.5 μm × 300 nm but with a highly detailed representation of the biological features.

To extend the reconstructions along the third direction beyond a 300-nm thickness, we imaged related areas from adjacent sections through tomography, and stacked their subsequent reconstructions into one single digital volume. The regions of interest were first mapped on a large scale (for instance with a 40 × 40 μm montage) to include distinctive features shared by the specimen sections that could be used as references for alignment. Such features generally have a characteristic dimension greater than the 300-nm sample thickness; they could be, for instance, nuclei, dendrites or very long mitochondria. Once the sequential maps were fully registered and aligned, the finer-scale localization of a specific region of interest can then be identified throughout all the specimen sections. In this work, serial reconstructions from up to five sequential tomograms were obtained.

We used an iterative base approach to obtain high-quality reconstruction for the individual tomograms (Phan et al., 2017). These were adjusted by means of a global affine transformation into the final volume of a serial reconstruction, as described by Phan et al. (2012). Discontinuities at the block interfaces were minor, suggesting phenomena such as nonlinear sample warping or material loss occurring during slicing and imaging were limited. The tomogram of spine H11 is shown in Movie 1.

Movie 1. Tomogram. Animated tomogram of the sample containing spine H11. 10.1523/ENEURO.0342-22.2022.video.1

### Data analysis

**Figure 1.**
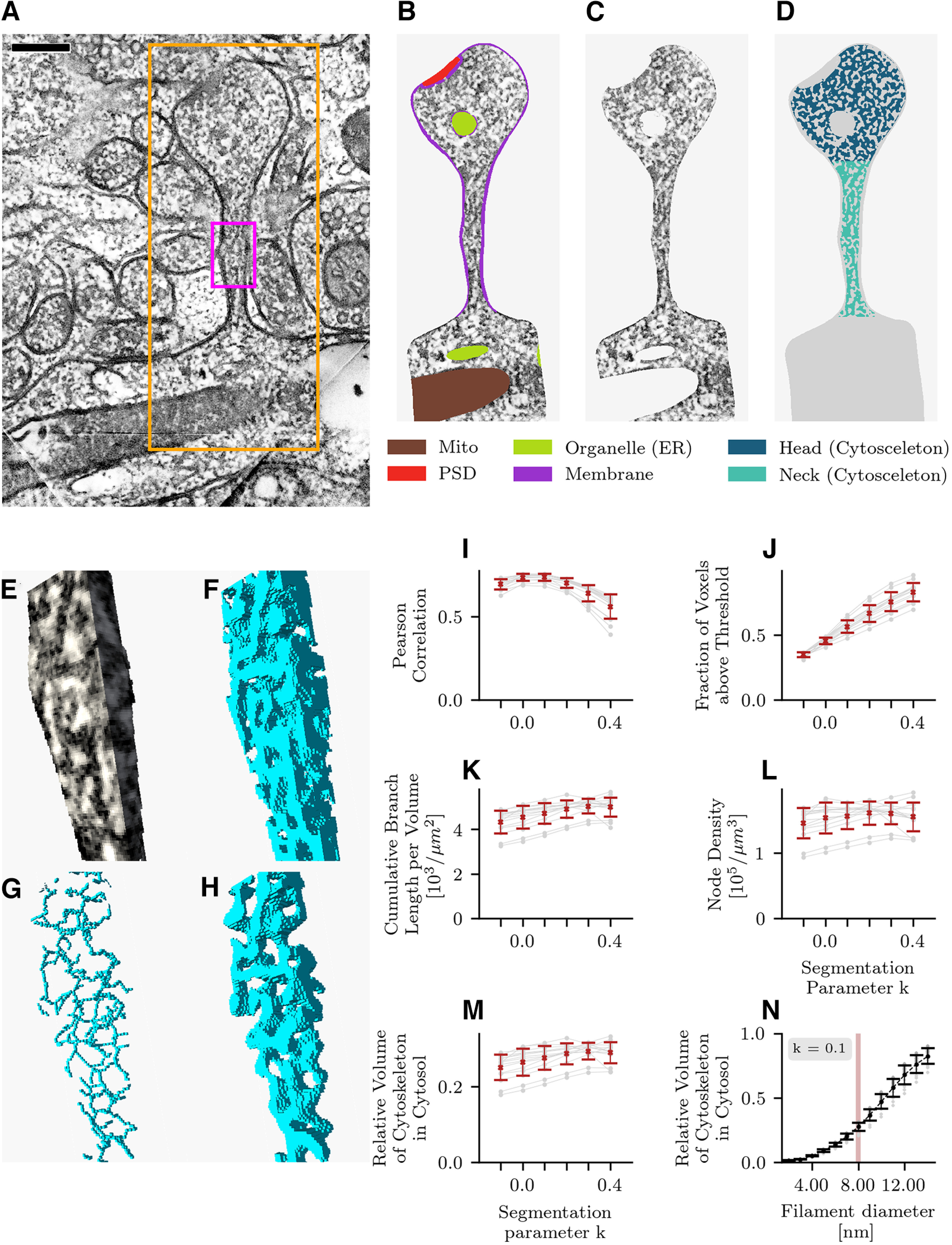
Spine segmentation. ***A***, Hippocampal EM section with dendritic spine H1 (large rectangle). Purple rectangle marks a section of the spine shown in ***E–H***. ***B***, Segmentation of spine H1 into cellular compartments (colors). ***C***, After masking the extracellular space, membrane, and organelles, electron-dense regions remain in the intracellular space. These dark fibrils correspond to putative actin filaments or actin-binding proteins. ***D***, After smoothing the tomogram with a Gaussian filter (Extended Data Fig. 1-1), image binarization separates the filaments from the background (water). ***E***, Three-dimensional visualization of the spine neck region marked in ***A***. ***F***, Binarization algorithm applied in 3 dimensions. ***G***, Extracting the topological skeleton of putative actin filaments. ***H***, Reconstructing putative actin filaments from the topological skeleton. ***I***, Correlation between absorption (luminance) and the result of the binarization algorithm as a function of the binarization algorithm parameter *k*. A positive value of *k* lowers the local threshold below the local mean value in the image. The threshold was made local to adapt to changing image statistics along the *z*-axis of the tomogram (Extended Data Fig. 1-2). ***J–M***, The binarization parameter *k* strongly affects the number of voxels above threshold that enclose the filaments (see ***D***, ***F***) but only weakly affects the resulting topological skeleton (***G***) as shown by the cumulative branch length (***K***), node density (***L***), and relative actin volume of the reconstructed cytoskeleton (***M***). ***N***, The relative volume of the reconstructed cytoskeleton as shown in ***H*** as a function of the reconstructed filament diameter. A full outline of the steps in the data analysis is presented in Extended Data Figure 1-3. Errorbars in I-N indicate mean and STD.

**Figure 2.**
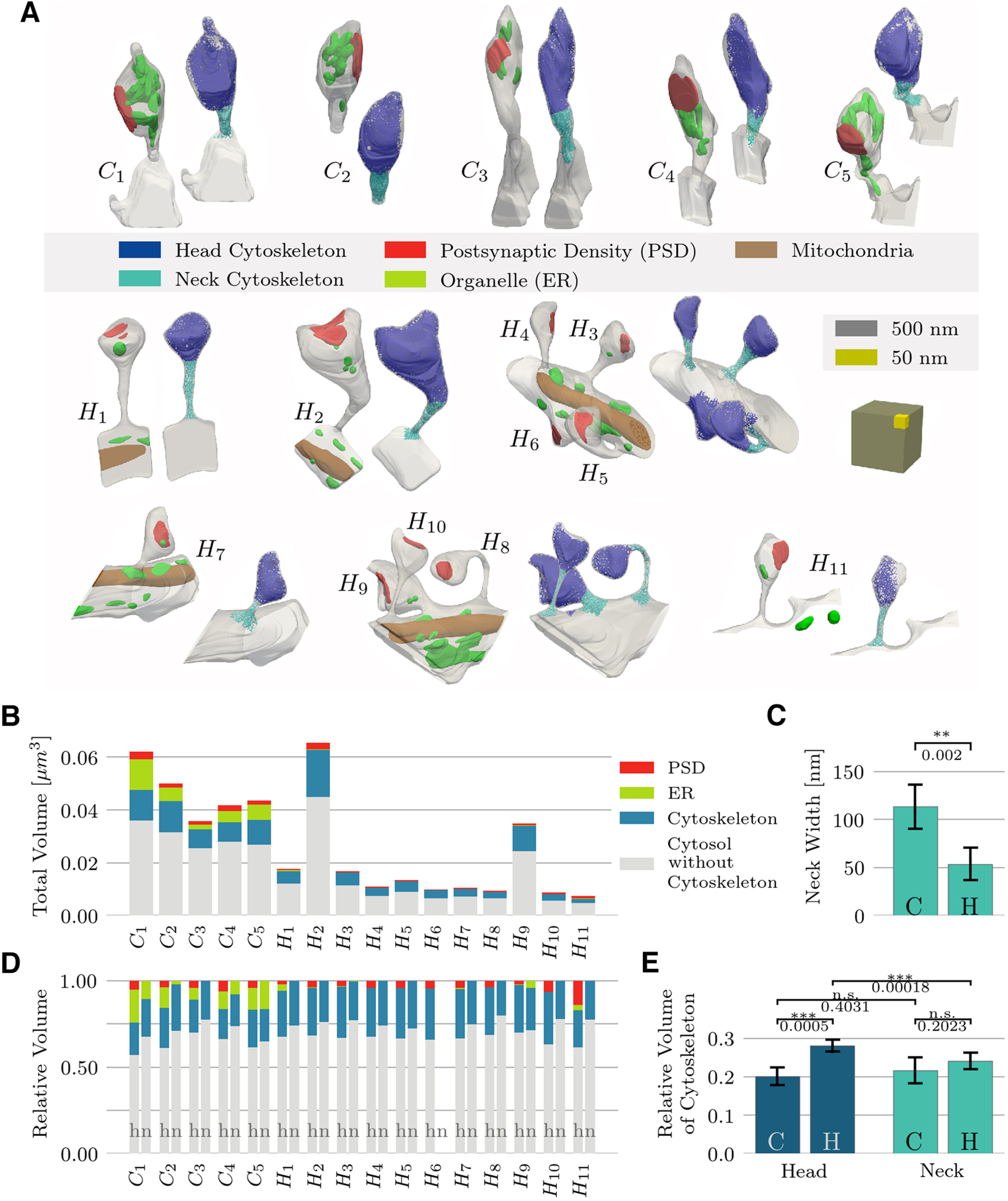
Spine volumes. ***A***, Analyzed spine population. Top row, Five spines from cerebellar Purkinje cells (C1–C5); lower part: 11 spines from hippocampal pyramidal neurons (H1–H11). Scale cube, gray: 500 × 500 × 500 nm^3^, yellow: 100 × 100 × 100 nm^3^. ***B***, Absolute volume of each spine, as stacked bar-plot, together with the individual components. ***C***, Mean and STD of the spine neck width, defined as the neck’s minimum diameter, for cerebellar Purkinje cells (C) and hippocampal pyramidal cells (H). ***D***, Relative volume of spine components, shown separately for the head and neck regions. ***E***, Comparison of the relative cytoskeleton volume in the heads and necks of cerebellar and hippocampal spines.

First, we identified several complete spines within the tomograms (Fig. 1*A*, Fig. 2*A*), which were segmented manually using the tomographic reconstruction software IMOD (http://bio3d.colorado.edu/imod). Within the manual segmentation step, the cellular membrane, the endoplasmic reticulum, the PSD and mitochondria were traced and stored as separate objects (Fig. 1*B*). After manual segmentation, we used the identified cellular components to mask regions outside the cellular membrane or within organelles. The remaining volume consisted predominantly of the cytosol and the cytoskeleton (Fig. 1*C*).

10.1523/ENEURO.0342-22.2022.f1-1 Extended Data Figure 1-1 Effect of filter width. Image noise was reduced by smoothing the tomogram with a Gaussian filter. The best results were obtained with a SD of 2 nm for the filter. Smaller SD s are not sufficient to eliminate noise and larger SD s do not preserve the topology of the cytoskeleton. Scale bar: 100 nm. Download Figure 1-1, TIF file.

10.1523/ENEURO.0342-22.2022.f1-2 Extended Data Figure 1-2 Local threshold binarization. A threshold is used to binarize the image into strongly and weakly stained voxels (see Extended Data Fig. 1-1). The mean luminance of a virtual slice through the tomogram varies along the *z*-axis. As the local luminance changes, the number of voxels above a fixed threshold changes, so we used a local threshold. The width of the gray area represents the size of the (sliding) window used for the computation of the local threshold. Download Figure 1-2, TIF file.

**Figure 3.**
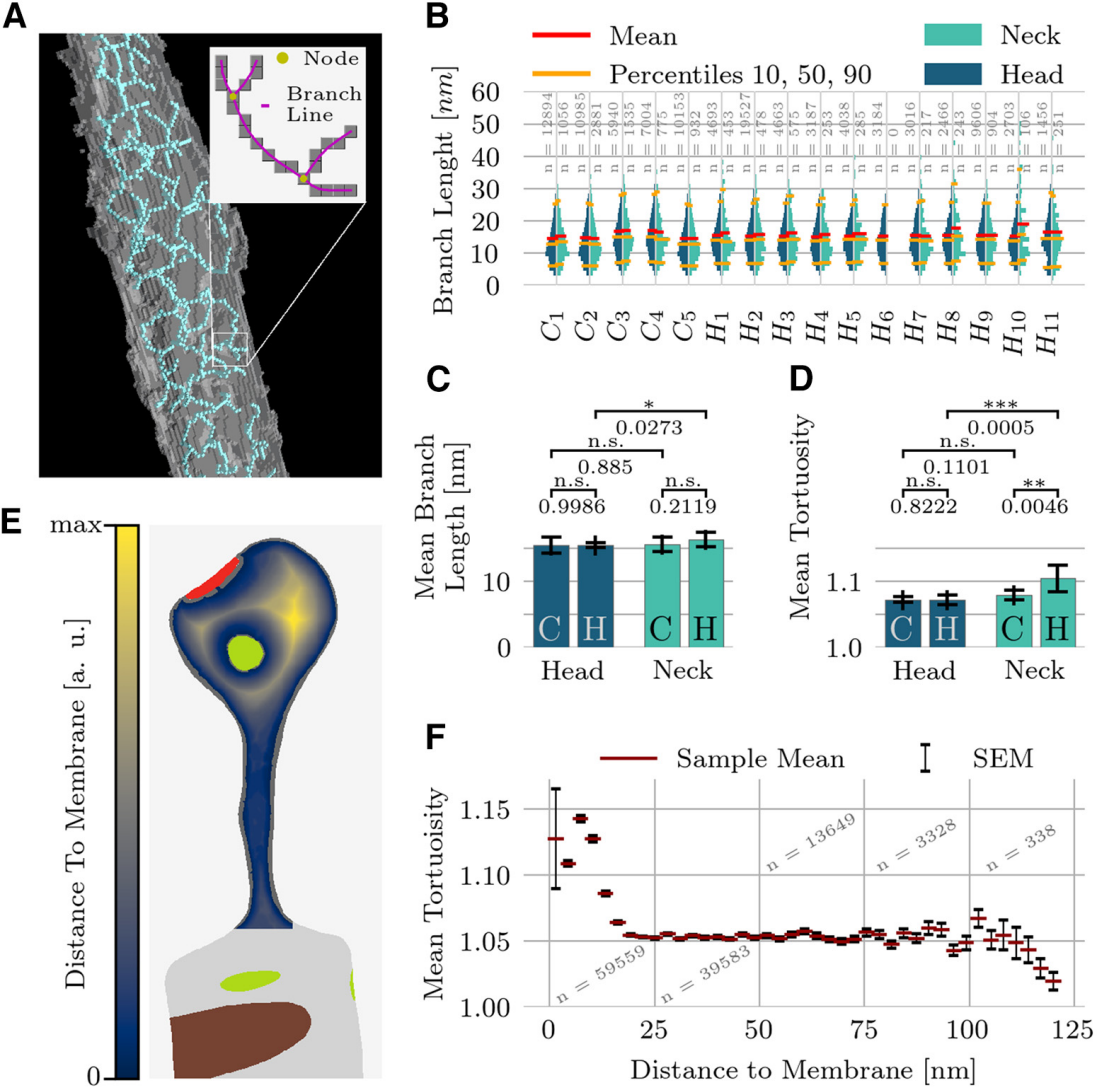
Length and bending of branches. ***A***, The topological skeleton is a set of connected voxels. A voxel connected to exactly two other voxels was considered to be part of a branch. A voxel connected to more than two other voxels was classified as a node (branching point). Voxels connected to only one other voxel were treated as branch ends. The branch length is computed as the arc-length of a smoothed curve through the voxels between two branching points (Materials and Methods). The tortuosity of a branch is defined as the ratio of the branch length to the Euclidean distance between the branch end points. ***B***, Branch-length distributions of the entire spine population, shown separately for the head and neck regions. Sample size shown in gray. ***C***, Pairwise comparison of mean branch length across hippocampal and cerebellar spines. Bar plots show the mean and STD of average branch lengths. ***D***, Pairwise comparison of mean tortuosity. Bar plots show mean and STD of average branch tortuosity. ***E***, Illustration of the normalized distance transform (DT), used here to measure the distance of any point within a spine to the spine membranes, which include the interfaces with the postsynaptic density and endoplasmic reticulum. ***F***, Tortuosity is increased close to the membrane and constant elsewhere. As hippocampal neck volumes are smaller and therefore have a higher surface/volume ratio this can explain the differences in ***D***.

10.1523/ENEURO.0342-22.2022.f1-3 Extended Data Figure 1-3 Coarse outline of the workflow. Following perfusion, a mouse brain is sectioned into thick (100 μm) slices using a vibrotome. Portions of interest are then punched out from brain slices (C stands for cerebellum, and H for hippocampus), before being processed through high pressure freezing and freeze substitution. This experimental protocol allows for an optimal sample preservation, which when combined with a multiple tilt EM tomography scheme on thin slices will provide high-quality reconstruction of the brain. Images ***A–C*** represent snapshots of the synapse corresponding to spine H11 taken at three different depths. The green stars (respectively, red circles) point to the presynaptic (respectively, postsynaptic) terminal. Note the overall quality of the synaptic vesicles (white arrow on panel ***B***) as well as the quality of the membrane definition (white arrow on panel ***C***) in this en face view. Once segmented out, the spine (red circles) is skeletonized and the resulting mesh, characteristic of the internal structure of the spine, is subsequently analyzed with the methods outlined and developed in this paper. Download Figure 1-3, TIF file.

In a second step, we reduced the image noise in the masked dataset by smoothing the tomogram with a Gaussian filter (STD for kernel 2.0 × 2.0 × 2.0 nm). Smaller STDs are not sufficient to eliminate noise and using a larger STD does not preserve the topology of the cytoskeleton, given that the smaller filaments like actin have a diameter of ∼8 nm (Extended Data Fig. 1-1). We then binarized the image using a local threshold (Sauvola and Pietikäinen, 2000), which divides the volumes into strongly stained regions (putative actin filaments) and weakly stained regions (fluid part of the cytosol consisting mainly of water; Fig. 1*D*,*F*).

For the computation of the local binarization threshold we used a window size of 20.0 nm, which corresponded to 10 voxels in the binned data set. The threshold was made local to adapt to changing image statistics along the *z*-axis of the tomogram; the luminance varied on a scale of tens of nanometers (Extended Data Fig. 1-2). The window size was also significantly larger than the Gaussian filter used for smoothing and larger than the minimum size of the structures under study. We chose the binarization algorithm’s segmentation parameter *k* to be 0.1. This choice lowered the local segmentation threshold below the local mean luminance of the imaging data and reduced the risk that local absorption fluctuations along the cytoskeleton would lead to filaments becoming disjoint after binarization. The segmentation parameter *k* affects the number of voxels above threshold in the binarized tomogram (Fig. 1*J*), yet the results of the structure analysis changed only little when that parameter was varied between −0.1 and 0.4, which indicated that the algorithm preserved the topology of the inferred graph (Fig. 1*K–M*). Moreover, the parameter choice *k* = 0.1 maximized the Pearson correlation between the raw data and segmented data (Fig. 1*I*). After binarizing the data, we again checked the boundary regions of the intracellular space. In regions where the manual segmentation was not precise, we masked, if necessary, remaining parts of the membrane in the segmented image datasets (Fig. 1*B*).

In a third step, we computed a distance transform on the binarized image and subsequently thinned out the distance transform to extract the topological backbone of the filament mesh.

For this purpose, we sorted the voxels by their distance transform value and proceeded to set voxels with lowest distance transform to zero based on the following topology-preserving rules: voxels were kept if their removal would “break” a filament or dissociate a filament from the volume boundary, or if the voxel lay at the end of a filament branch. This procedure yielded lines of connected voxels representing the structure of the putative actin cytoskeleton (Fig. 1*G*). A voxel connected to exactly two other voxels was considered to be part of a branch. A voxel connected to more than two other voxels was classified as a node (branching point). Voxels connected to only one other voxel were classified as branch ends. In the 3D stack, some filaments apparently crossing adjacent volume subsections did not correspond to a continuous voxel sequence; the implication is that the algorithm introduces some artificial branch cuts, which were excluded from further analysis. Structures that were smaller than the smallest expected structures (4 nm) were removed, which almost exclusively removes small branch ends. Such “stubs” were quite possibly the result of imaging noise. For further analysis, we converted the filamentous structure into a NetworkX python graph object (Hagberg et al., 2008; version 2.4). A summary of the procedure described above can be seen in Movie 2. While supervised machine learning algorithms for segmentation (Ronneberger et al., 2015) exist, these require a ground truth for training, which can only be derived from manual segmentation. Our goal was to derive results that are relatively independent of how the volume is segmented. The procedure of local threshold binarization and thinning comes close to achieving this goal and preserves the topology of the cytoskeleton throughout high-absorption and low-absorption regions and is relatively independent of how the volume is segmented.

Movie 2. A procedure to detect the topological structure of the actin cytoskeleton in dendritic spines. The video illustrates the individual steps to extract the topological structure of the actin cytoskeleton, first applied to a 2D image of artificial test data, and then applied to a 2D slice of an electron tomogram. Extending the method to 3D is straightforward, and not illustrated here. Reconstructing the topology within a 2D slice of the 3D EM tomogram, as shown here, leads to a lower connectivity and a higher number of branch ends than if one traces the filaments also along the third axis pointing into the plane, as we did in this study. 10.1523/ENEURO.0342-22.2022.video.2

#### Volume and neck width

We estimated the volume of membrane-bound organelles (mostly endoplasmic reticulum) and postsynaptic density (PSD) by summing the voxels belonging to each cellular component in the manually segmented masks (Fig. 1*B*). Furthermore, to estimate the volume taken up by the filaments in the intracellular space (confined to the cytosol subvolume), we convolved the extracted topological skeleton of the filaments (Fig. 1*G*) with a Gaussian filter (σ = 4 nm) and counted all voxels whose values were above α e^−0.5^, where α = 1/(2 π σ^2^). Applying this threshold value reconstructs a volume that corresponds to F-actin, which has a diameter of 8 nm (Fig. 1*H*,*N*), so this measure assumes that the putative cytoskeleton is predominantly actin. The minimum diameter along the spine neck was manually measured in ParaView (Ahrens et al., 2005).

#### Branch length and tortuosity

Each branch consists of a set of connected voxels. From each such set of voxels, we extracted a smoothly bending curve that connected the two nodes as follows: start and endpoint of each curve were the fixed positions of adjacent nodes. When the algorithm classified more than one voxel as part of the same node, the node position was taken to be the center of mass. The branch curve was then determined by a weighted average [sliding weights: (1,2,3,2,1)/9] of the positions of the surrounding branch voxels (Fig. 3*A*). The branch length was computed by the arc-length integral along the branch curve. For each of the 16 spines, we separately computed the branch-length distributions in the two main spine subdomains, the head and the neck (Fig. 3*B*). In addition, we computed the tortuosity as the ratio between the branch length and the Euclidean distance between the nodes of that branch. On a finer spatial scale, we examined the mean tortuosity (at ∼3-nm bin-size resolution) with respect to the distance to the closest membrane (Fig. 3*F*). For this purpose, we computed the distance transform of the intracellular space with respect to the spine boundary (Fig. 3*E*; cf. Felzenszwalb and Huttenlocher, 2004). Any organelles present, which were characterized by being devoid of cytoskeletal elements, were considered part of the boundary. A branch’s distance to the closest boundary was computed as the distance transform value of the branch’s center of mass.

#### Node distribution

**Figure 4.**
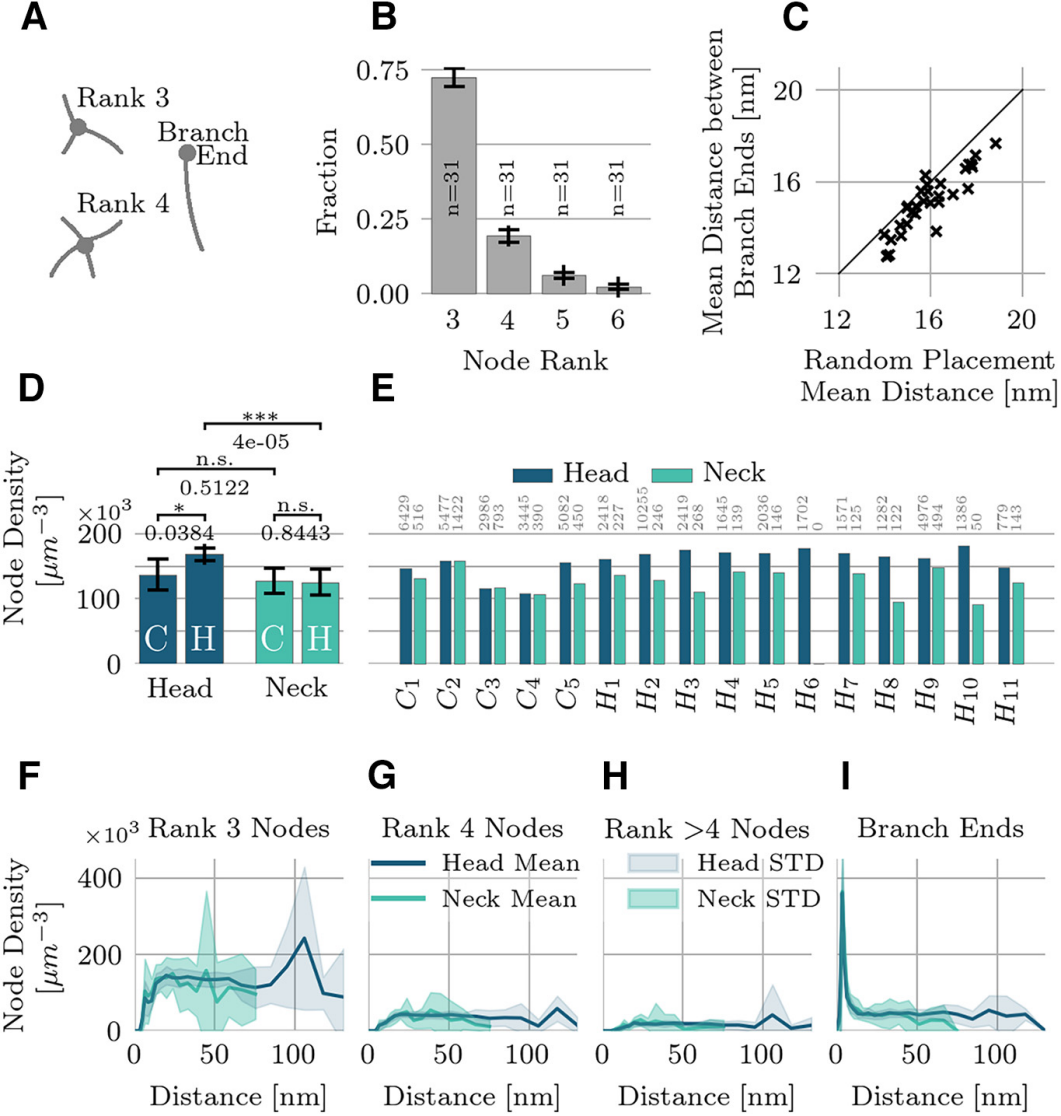
Density and spatial distribution of actin nodes. ***A***, The node rank counts the number of branches that meet at anode. ***B***, Node rank prevalence (for rank >2), calculated for the entire population (mean and STD). ***C***, Nearest neighbor analysis of branch ends within 10 nm of a membrane. The mean nearest neighbor distance between branch ends is slightly smaller than if they were randomly placed (*p* = 0.02). ***D***, Pairwise comparisons of the rank-3 node density. Bar plots show mean and STD. The surface-to-volume ratio is a good predictor of the average node density within a spine (Extended Data Fig. 4-1). ***E***, Rank-3 node density for the entire population, shown separately for head and neck regions. Sample size indicated by gray numbers. ***F*–*I***, Spatial density of rank 3 (***F***), rank 4 (***G****)*, nodes of rank >4 (***H***), and branch ends (***I***) as a function of their distance to the nearest membrane given by the distance transform (Fig. 3*E*). The node density is constant throughout the inner regions and drops near the boundary. Branch ends are preferentially found close to the membrane. Lines indicate mean values and colored regions the STD.

The node rank of a node counts the number of branches that meet at the node (Fig. 4*A*). For rank-3 nodes, we estimated the mean density inside the accessible intracellular space (ignoring organelles and PSD) in all domains. Then we spatially resolved the density of nodes of different ranks within the intracellular space for all spines (Fig. 4*F–I*). For this purpose, we mapped the nodes onto the scalar-field returned by the distance transform (within the intracellular space taking into account organelles and PSD; see Fig. 3*E*), which allowed us to evaluate how the nodes were distributed within the intracellular volume of the spines. We quantified these distributions in terms of the node density relative to the total distance to the membrane. Finally, we analyzed the distribution of branch ends within 10 nm of a membrane surface, corresponding to the strong peak in the branch end distribution (Fig. 4*I*). For each branch end in this region, we searched for the nearest neighboring branch end inside that region and measured the Euclidean distance between them. We compared the distribution of branch end distances to that of randomly placed branch ends. Specifically, we placed an equivalent number of points at random within the boundary region and then analyzed the resulting distribution of nearest neighbor distances.

10.1523/ENEURO.0342-22.2022.f4-1 Extended Data Figure 4-1 Correlation between surface-to-volume ratio and node densities. The surface-to-volume ratio is a good predictor of the average node density within a spine. Download Figure 4-1, TIF file.

#### Branching angles

**Figure 5.**
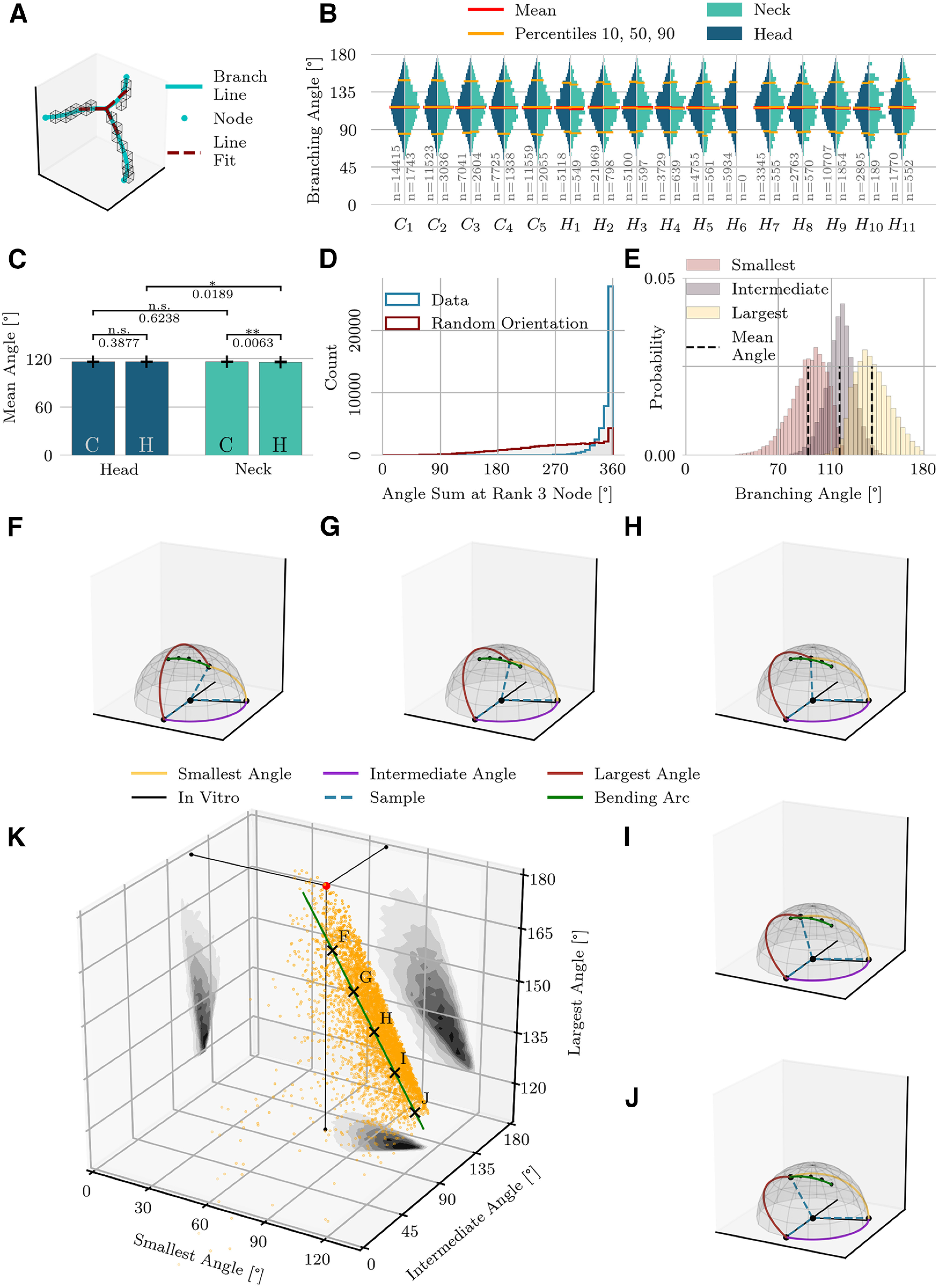
Branching angles. ***A***, Illustration of a rank-3 node. Voxels of topological skeleton are indicated by boxes. Smoothed branch lines are shown in blue. A line fit (red dotted line) approximates the orientation of the branches. The angles between the red lines represent the branching angles. ***B***, Distribution of the branching angles of the entire population, shown separately for the head and neck regions. Sample sizes shown in gray. ***C***, Pairwise comparison of mean branching angles. Bar plots show the mean and STD of average branching angles (mean and STD are computed on average values for individual spine domains). ***D***, The sum of the three branching angles at a rank-3 node tends to be close to 360°, indicating that the branches nearly lie in a plane. When the branching angles are random, in contrast, the sum of angles is often lower. ***E***, Distributions of angles at rank-3 nodes, shown separately for smallest, intermediate and largest angles. Mean angles deviate from *in vitro* actin branching angles. ***F*–*J***, Branching patterns at the points indicated by matching letter labels in ***K***. A 70°−110°−180° branching-pattern is shown for comparison by the black solid lines. The intermediate angle (110°) remains fairly stable across these examples, while a single branch changes its position as indicated by the green arc. ***K***, 3D distribution of the three measured branching angles at rank-3 nodes. The red dot indicates the planar 70°−110°−180° model that corresponds to *in vitro* actin branching. The first principal component of this distribution, representing 57% of the total variance, is shown as a green line segment.

The angle between two branches at a node was determined by approximating the tangential vectors to the filaments by the principal component of the first three points along each branch curve, starting with the node position (Fig. 5*A*). In three dimensions, the branches at a rank-3 node do not, in general, lie in a plane, but form a tetrahedron. We sorted the angles into smallest, intermediate, and largest. Then we transformed the branch vectors at rank-3 nodes to a set of standardized coordinates that is as close to “planar” as possible. The vector making the largest angle with the other two is set to (x,y,z) = (−1,0,0). An azimuthal plane is defined by this first vector and the one making the intermediate angle with the first vector. The third vector is then chosen to have a positive elevation above the plane.

#### Branch orientations

**Figure 6.**
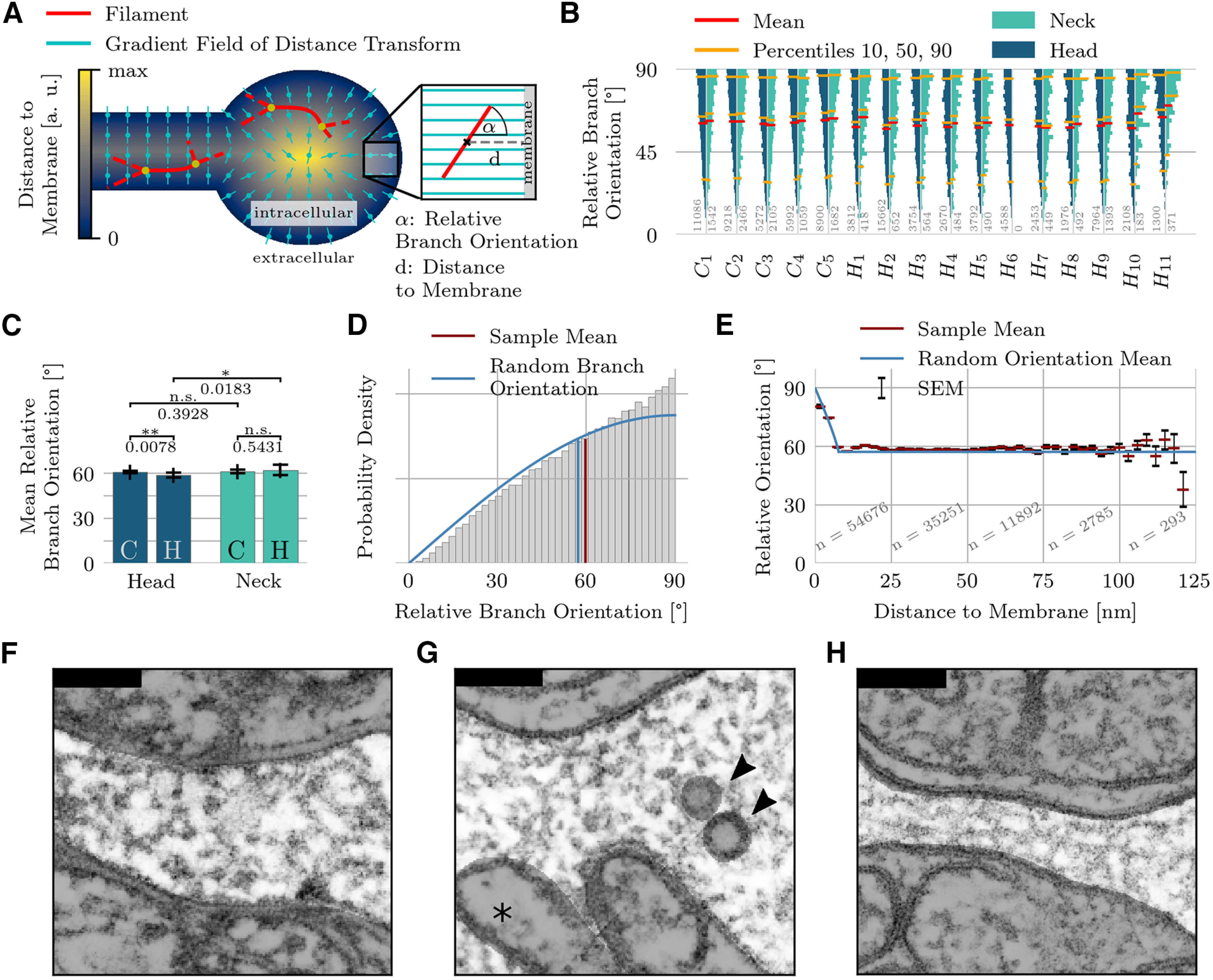
Branch orientations. ***A***, The 3D orientation of a branch is given by the angle between the branch’s tangent vector and the reference vector field given by the gradient of the distance transform on the intracellular space. The distance to the membrane is always measured relative to a branch’s center of mass. ***B***, Distribution of the relative branch orientations of the entire population, shown separately for head and neck regions. Sample size indicated by gray numbers. ***C***, Pairwise comparison of mean relative branch orientations. Bar plots show mean and STD of the average branch orientations of individual spine domains. ***D***, Full distribution of relative branch orientations pooled over all spines. Angles close to 90° are overrepresented compared with a distribution of random branch orientations in free space. ***E***, Detailed analysis of mean relative branch orientation as a function of the distance to the membrane (Fig. 3*E*). The SEM is indicated by black bars. If one treats the branches as rigid rods, then branches in the immediate membrane vicinity must run parallel to the membrane, although orientations are generally random. ***F***, ***G***, Filaments in spine necks are typically not aligned with the neck’s main axis, as shown by two examples (***F***, C3; ***G***, H9). Head of H9 contains organelles (arrowheads) and the dendrite mitochondria (asterisk). ***H***, Long and thin spine necks do, however, constrain the filament orientation, as demonstrated by the H11 spine. The neck’s minimum diameter is 91.0 nm in C3, 86.5 nm in H9 and 35.1 nm in H11. Scale bars in ***F***, ***G***: 100 nm.

To analyze the branch orientation relative to the spine geometry, we computed the gradient field of the distance transform with respect to the full cytosol volume (organelles and PSD are excluded). This distance transform reaches its minimum at the cell membrane and its maximum at the center, so the gradient vectors point away from the membrane toward the center of the volume. For each branch, we computed the angle between the distance transform gradient and the branch (measured by the central line through three adjacent branch points) and averaged this angle across all points on the branch (Fig. 6*A*). This results in one orientation measurement per branch connecting two branching points (nodes). The mean orientation value along each branch was computed because tortuous branches change their local orientation as a function of position. From these measurements we computed the distribution of the branch orientations in all spine volumes. On a finer spatial scale (at ∼3-nm bin-size resolution), we examined the mean branch orientation with respect to the distance to the closest membrane (Fig. 6*E*) whereby the position of each branch was taken to be its center of mass.

#### Elementary loops

**Figure 7.**
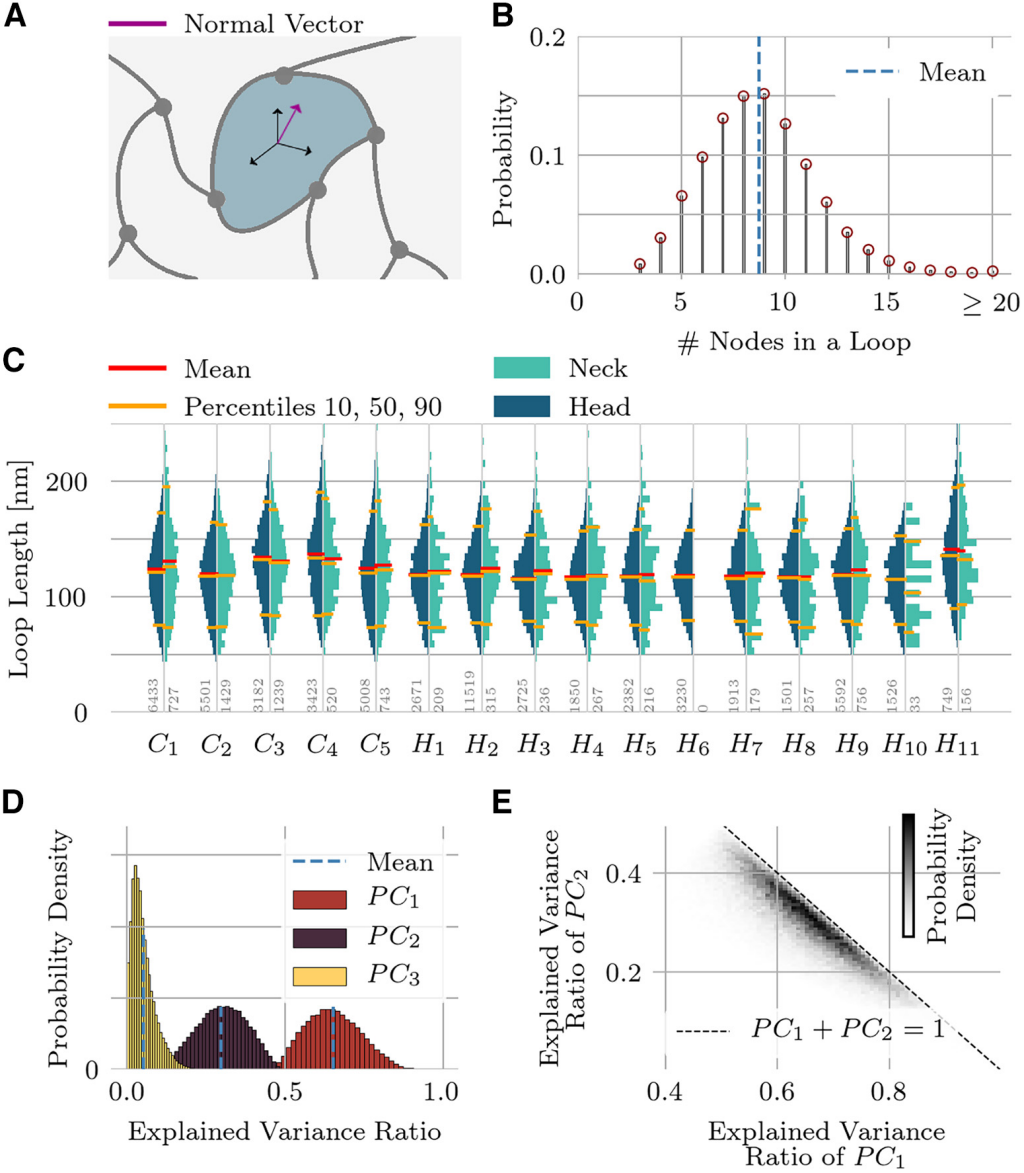
Elementary loops. ***A***, An elementary loop through a branch within the network mesh is defined as the shortest cycle in a graph that contains that branch. ***B***, Distribution of the number of nodes/branches in an elementary loop. ***C***, Loop-length distributions for the entire spine set, shown separately for the head and neck regions. ***D***, A principal component analysis dissects the spatial arrangement of nodes inside an elementary loop. The first two components account for 95% of the total variance, which implies that elementary loops are predominantly planar. ***E***, The first two principal components plotted against each other, shown as a density plot for all loops in the set. The dashed line corresponds to perfect planarity, i.e., vanishing third principal component.

A loop is an elementary cycle in the graph (Fig. 7*A*). To find all loops going through a certain node we considered the subgraph that lacks that particular node. Dijkstra’s shortest path algorithm (weighted by branch length), as implemented in NetworkX (Hagberg et al., 2008), finds the shortest path between all pairs of nodes originally connected to the chosen node, provided such a path exists. The final path length (loop circumference) of the shortest loop was obtained by adding back in the lengths of the two branches that connect to the node that was initially removed. This procedure was repeated for all nodes, yielding a set of loops (after eliminating duplicates). We then analyzed the loop circumference distribution for all spines and the distribution of node numbers in a loop. For every loop, we also performed a principal component analysis (PCA) to determine the best fit of a plane to the points of the loop.

### Statistical analysis

Pairwise comparisons are shown in Figures 2*C*,*E*, 3*C*,*D*, 4*D*, 5*C*, and 6*C*. We divide the data into four different domains; hippocampal heads (11 samples), hippocampal necks (10 samples), cerebellar heads (five samples), and cerebellar necks (five samples). Statistical measures are computed in each domain sample. The figures listed above show the mean value and the STD of the neck width (no head domains), the relative volume of putative actin, average branch length, average branch tortuosity, node density, average branching angle at rank three nodes and average branch orientation. We also report the statistics of these measures across domains. For example, we first measured the average branch length in individual hippocampal spine heads and then computed the mean values and STD of the average branch length across all 11 hippocampal spine heads. Finally, we computed *p*-values with a Welch’s *t* test to quantify differences between domains types (e.g., hippocampal heads vs cerebellar heads). The normality of the distributions was always tested with a Shapiro–Wilk test.

In Figures 1*I–N* and 4*B*, we provide the mean and STD for all measured properties in the red and black bars. Moreover, in Figure 4*F–I*, we show mean values (solid lines) within a certain (normalized) distance bin and the STD as colored area around the mean values. Here we differentiated between head and neck but not between cell types. Finally, Figures 3*F* and 6*E* show mean relative branch orientation within a certain distance to the membrane and the SEM. All spines and domains were pooled together in Figures 6*E* and 3*F*.

### Notation

We use the term “domain” to denote the head or neck of a pyramidal or Purkinje cell spine. For instance, the head of cerebellar spine 1 denotes one specific spine domain.

### Code accessibility

A Python-notebook with the work-flow to extract the topological structure of the actin cytoskeleton in dendritic spines from EM tomography data will be available on GitHub on publication (https://github.com/feblmu/cytoskeleton).

An outline of the steps in the data analysis is presented in Extended Data Figure 1-3.

## Results

Using multitilt serial section electron microscopic tomography on high-pressured frozen samples, we identified and imaged 16 dendritic spines from mouse hippocampal pyramidal neurons and cerebellar Purkinje cells at a nominal resolution of 0.4 nm (Figs. 1, 2*A*; Movie 1), which revealed the highly branched, filigree architecture of spine interiors. After voxel-averaging and filtering to reduce imaging noise, we reconstructed the full three-dimensional geometry of the cytoskeleton at 2-nm resolution. This cytoskeleton is composed of densely branching actin and cross-linking proteins. *In vitro*, actin filaments branch out extensively in spine heads (Fifková and Delay, 1982; Hotulainen et al., 2009; Korobova and Svitkina, 2010; Lei et al., 2016), while actin in spine necks exhibits a combination of branched and some longer filaments that tend to align with the neck’s longitudinal axis (Korobova and Svitkina, 2010). Spines in cell culture, however, are not subject to the same mechanical stresses and electrostatic forces as in the living animal. Our aim was to quantify the geometry of the cytoskeleton of these *in situ* spines at high resolution, given the geometry’s implications for the electrochemical compartmentalization, mechanical stability, and force generation in spines.

### Volume statistics

To start our analysis of the 3D fine structure of dendritic spines, we measured the size of the 16 specimens, 11 hippocampal and five cerebellar spines. Spine H6 was stubby without an identifiable neck (Fig. 2*A*). Their volumes range from 0.01 to 0.07 μm^3^ (Fig. 2*B*). The mean volume of hippocampal spines was 0.02 ± 0.02 μm^3^; the volume of cerebellar spines was 0.05 ± 0.01 μm^3^. These values are consistent with reports by Bartol et al. (2015) on hippocampal spines and Harvey et al. (2005) on proximal dendritic spines of Purkinje cells. For both cell classes the postsynaptic density (PSD) size and spine-head volume were strongly correlated (hippocampal spines: *r* = 0.92, cerebellar spines: *r* = 0.85), again in agreement with the literature (Arellano et al., 2007; Bartol et al., 2015). The width of the spine necks, defined as the minimum diameter along the neck, ranged from 91.0 to 145.9 nm (113 ± 23 nm, mean ± STD) in Purkinje cells and from 30.4 to 86.5 nm (54 ± 17 nm, mean ± STD) in hippocampal pyramidal cells (Fig. 2*C*). These values are comparable with the thinnest spine necks found in other studies of rat Purkinje cells (Napper and Harvey, 1988) or mice pyramidal cells (Tønnesen et al., 2014).

Next, we measured the volume occupied by the putative actin-cytoskeleton inside the dendritic spines. The reconstructed cytoskeleton comprised between 17% and 23% (20 ± 2%, mean ± STD) of the volume in the heads and between 18% and 27% (22 ± 3%, mean ± STD) in the necks of cerebellar Purkinje cells (difference not statistically significant; Fig. 2*D*,*E*). In contrast, hippocampal spine heads showed a greater volume taken up by the cytoskeleton when compared with hippocampal spine necks, ranging from 26% to 30% (28 ± 1%, mean ± STD) in the spine heads to ranging from 20% to 28% (24 ± 2%, mean ± STD) in the spine necks (*p* = 0.0002). The difference in the cytoskeleton fraction in spine heads was significant across cerebellum and hippocampus (*p* = 0.0005), whereas we did not find a difference between the spine necks of both cell types. The cytoskeleton volume analyses assume that all filaments have a diameter of 8 nm.

Adding together the volumes taken up by membrane-bound organelles, PSD and the putative actin cytoskeleton, we found the pooled volume of these structures summed to 37 ± 5% in cerebellar heads, 29 ± 5% in cerebellar necks, 34 ± 4% in hippocampal head and 25 ± 3% in hippocampal necks. This analysis indicates a lower proportion of water in the spine heads in comparison to necks (cerebellum: *p* = 0.04, hippocampus: *p* = 2.3 × 10^−6^), but no significant difference in the water proportion between Purkinje cells and pyramidal cells in either domain.

### Branch length and tortuosity

When shielded from external forces and away from branch points, actin can form long, straight filaments. There are both linear and branched actin filaments in spine heads and necks; they are more frequently linear in necks and predominantly branched in heads (Lei et al., 2016). Does this hold *in situ*, too?

To answer this question, we first computed the individual branch length distribution for all spine domains (Fig. 3*B*). The mean branch length (arc length between two branching points) across all spines was found to be 15.4 ± 8.0 nm (mean ± STD) and 80% of all branch lengths lay between 6.6 and 25.9 nm. Next, we searched for variability in the branch length distributions between brain regions and between head and neck. The mean branch length was similar in all domains, ranging from 14.6 to 18.9 nm (Fig. 3*C*). Only in hippocampal necks did we find a slightly higher mean branch length on average than in hippocampal heads (average over mean values: 16.5 vs 15.5 nm, *p* = 0.03). Next, we normalized the individual branch-length distributions of each domain (Fig. 3*B*) by the median branch length of that domain. For most pairwise comparisons, we found no significant difference between the resulting distributions (KS tests, *p* > 0.05 for 800 out of 930 pairs).

To capture the intrinsic geometry of each branch, we measured its tortuosity, defined as the ratio between arc-length and the straight-line distance between branch end points (Fig. 3*A*, inset). A Welch’s *t* test showed a significant increase of the average tortuosity in hippocampal necks (Fig. 3*D*). The average tortuosity of all domains ranged from 1.07 to 1.15. We found that a domain’s volume was a good predictor of the mean tortuosity of the branches, with smaller domains (mainly hippocampal spine necks) having higher tortuosity (Spearman correlation between volume and tortuosity: −0.82). We also examined how the measured tortuosity changes with the distance to the nearest membrane. To answer this question, we calculated the distance transforms between points of interest and the entire spine surface, including interfaces with the endoplasmic reticulum and the postsynaptic density (Fig. 3*E*). The center of mass of each branch was then mapped onto this distance field. For all spines, the tortuosity was significantly higher close to the membrane but took on a constant value (∼1.05) elsewhere (Fig. 3*F*). As small volumes have a higher surface to volume ratio, the fact that many filaments in a small volume are inherently close to the surface leads to a negative correlation between volume and tortuosity.

Tortuosity can be expressed in terms of an isosceles triangle: the base of this triangle represents the Euclidean distance between the end points of a branch, and the two equal sides sum up to the total path length of the branch. A tortuosity of 1.05 corresponds to an angle between the base and the sides of 17° (whereas 1.15 corresponds to 30°). Therefore, the measured tortuosity indicates a considerable bending of the filamentous structure. Moreover, the increased tortuosity close to the membrane is consistent with the membrane exerting additional mechanical stress on the putative actin cytoskeleton.

In summary, this analysis showed that filaments in dendritic spines *in situ* form a dense and tortuous mesh subject to considerable deformation and exhibiting no long-range organization. The findings also support the view that the mechanisms behind the regulation of the cytoskeleton architecture are similar or even identical in cerebellar and hippocampal spines.

### Nodes

We counted the number of branches connecting to all individual nodes of the mesh. Neglecting branch endings (rank-1 nodes), ∼72% of nodes connect three branches (rank-3 nodes), ∼20% are rank 4, roughly 6% link five branches, and the remaining 2% are of rank 6 or higher (Fig. 4*B*). We did not observe significant differences in these numbers when comparing hippocampal and cerebellar spines nor when comparing heads versus necks (18 out of 24 pairwise comparisons were not significant based on a Welch’s *t* test at a significance level of 0.05).

Node densities (node rank three and higher) varied between 91 × 10^3^ and 181 × 10^3^ per μm^3^ (Fig. 4*E*) and were higher in spine heads (mean node density 1.59 × 10^5^ per μm^3^) compared with spine necks (mean node density 1.18 × 10^5^ per μm^3^). A Welch’s *t* test found a significant difference between hippocampal spine heads and necks but no significant difference in Purkinje cells (Fig. 4*D*), consistent with the relative actin volume (Fig. 2*E*).

We refined the spatial analysis to ask whether nodes are preferentially positioned close to the spine’s surface, far from it, or are more-or-less uniformly distributed. We computed the distance of the nodes to the surface using the distance transform as shown in Figure 3*E*. Next, we compared the node density of all spines as a function of the total distance to the closest membrane for rank-3, rank-4, rank->4 nodes and branch ends (Fig. 4*F–I*). Node densities in head and neck domains were generally similar, but the node density dropped in the vicinity of the membrane, whereas the branch-end density increased. The boundary region with lower node density was comparable in size to the mean branch length, which suggests that close to the boundary filaments form connections with the membrane rather than with other filaments. Apart from the boundary region, the nodes populate the available volume homogeneously.

We found that the surface-to-volume ratio is a good predictor of the average node density within a spine (Extended Data Fig. 4-1, Pearson correlation *p* = 2.76 × 10^−6^). Because of their small diameter, the surface-to-volume ratio is higher in thin spine necks when compared with heads, leading to a stronger contribution of the membrane region in thin necks. The larger surface-to-volume ratio in necks likely explains the difference in the node densities between necks and heads (Fig. 4*D*,*E*), consistent with the average node density dropping more strongly in thin spine necks than in spine heads. Moreover, because of the increased surface by the many intracellular organelles, cerebellar spine heads have on average a larger surface-to-volume ratio than hippocampal spine heads (Extended Data Fig. 4-1), which is once again reflected in the difference between pyramidal and Purkinje cells (Fig. 4*D*,*E*). This observation is consistent with the hypothesis that a common mechanism regulates the spine cytoskeleton across cell types and domains.

While the branch nodes are uniformly distributed, the branch ends alongside the membrane might not be, particularly in the vicinity of the membrane. A recent report reveals that actin organizes the clustering of the transmembrane adhesion molecule CD44, both on the scale of a few nanometers to hundreds of nanometers (Sil et al., 2020).

To detect signs of clustering in our data, we analyzed the distribution of putative branch ends that occurred within 10 nm of the membrane. We compared this distribution to a reference model, for which brand ends were randomly distributed (Clark and Evans, 1954). If the average distance to the nearest neighboring branch end is shorter than in the reference, then the branch ends aggregate; on the contrary, if the average distance is larger, than the branch ends are dispersed; in the extreme case, the branch ends align to a regular grid (Clark and Evans, 1954).

The average nearest neighbor distance across all spine domains was 15.0 ± 1.2 nm (mean ± SEM) ranging from 12.7 to 17.6 nm (Fig. 4*C*). We found no difference in the mean nearest neighbor distance between heads and necks or cerebellar and hippocampal cells. The mean distance was slightly smaller (*p* = 0.02, Mann–Whitney *U* test) than the average nearest neighbor distance found for randomized distributions 15.7 ± 1.3 nm (mean ± STD) ranging from 14.0 to 18.8 nm. Hence, there is a weak tendency for branch ends to aggregate.

### Branching angles

Most branching points (72%) were rank-3 nodes (Fig. 4*B*), which could be a signature of the Arp2/3 complex. This complex is known to initiate growth of a new actin filaments from a preexisting filament, thereby creating a rank-3 node. A previous study had found Arp2/3 in all the heads and 30–40% of the necks of hippocampal spines (Korobova and Svitkina, 2010), so we investigated the possible relationship between rank-3 nodes in our data and Arp2/3-mediated branching of actin.

*In vitro* studies report actin branching angles close to 70° (Blanchoin et al., 2000; Pollard et al., 2000). Depending on specimen, preparation, and data analysis, the branching angles vary between 30° and 70° in Rigort et al. (2012) or as little as 70 ± 7° (mean +/– SEM) in a study with 48 branch points (Mullins et al., 1998). To address whether these values also hold *in situ*, we compiled the statistics on all three angles of all rank-3 nodes for each spine domain (Fig. 5*B*). Contrary to expectations, these distributions showed no peaks at 70°, 110°, or 180°. An analysis of average branching angles revealed only small differences between heads or necks or between cell types (Fig. 5*C*); therefore, we combined the statistics for further analysis.

We investigated whether the branching angles at rank-3 nodes could be a sign that the cytoskeleton is bending under mechanical pressure, which might explain the deviations from the predicted 70° branching angle. We had similarly attributed the data on tortuosity (Figs. 3, 4) to mechanical stress, and a previous study has found that branching of actin filaments is sensitive to mechanical deformations, with Arp2/3 preferentially initiating new branches on the convex side of bent actin filaments (Risca et al., 2012).

In the standard model of actin assembly, Arp2/3 mediates the binding of new branches to existing branches at an angle of 70° (Blanchoin et al., 2000), with both filaments lying in the same plane. But if the structure starts bending under mechanical stress, planarity need no longer be fulfilled, and the three angles between the filaments at a rank-3 node will sum to <360°. We measured the angle sum for all rank-3 nodes and compared the results to the case of random branch orientations. Across all rank-3 nodes of all spines and domains the branching of actin filaments *in situ* was closer to planar, on average, than for randomly oriented branches (Fig. 5*D*). Indeed, in many cases, the filaments bent within the plane. To further analyze the distribution of branching angles we sorted the three branching angles at rank-3 nodes from small to large. The distribution of largest angles had a mean value 140.8°, less than the 180° expected from a straight-line filament from which a side filament branches off. Moreover, the smallest angle (mean 92.4°) was larger than the expected 70° angle; only the intermediate angle (mean 116.2°) was close to the expected 110° angle. For comparison, if the branching angles from a rank-3 node were completely random, the three angles, ordered according to size, would have mean values of 53.6°, 95.3°, and 121.1°. Not only are the real branching angles larger than expected from a random hypothesis, they are less variable (STD 14.3°, 11.0°, 13.9° for the measured angles vs 24.4°, 27.1°, 30.9° for random angle branching).

Figure 5*K* displays the full three-dimensional distribution of the branching angles. A red dot in Figure 5*K* references the planar 70°−110°−180° model, allowing one to visualize the deviations of the data’s rank-3 configurations from the standard model. The axis shown in green accounts for 57% of the total variance in the distribution of branching angles. For selected, labeled points along the green principal component axis, we display the three-dimensional branching pattern (Fig. 5*F–J*). The intermediate angle in these five examples lies close to 110°. The complementary angle, found to be 70° *in vitro*, on the other hand, is frequently larger *in situ*. As one traverses the principal component axis from F to J, the points in the branching angle distribution move away from the standard model. In the corresponding sequence of example branching patterns, this causes one of the branches to move out of the horizontal plane. This moving branch traces out the green arc shown throughout Figures 5*F–J*. If one treats the largest angle (red arc in the examples) as a bending within a single filament, then a daughter branch splits off from the convex side of that bending.

In summary, we found that branching angles at rank-3 nodes are highly variable, which is possibly a consequence of mechanical deformations. In line with the prior quantitative analyses, angle distributions were fairly consistent across cerebellar and hippocampal spines and across spine necks and spine heads.

### Branch orientations

According to the classical view, actin filaments in spine necks have a greater tendency to run parallel to each other along the neck, but take on random directions in spine heads (Lei et al., 2016). As our preparation allows us to determine the 3D orientation of every filament, we decided to test whether these branches follow any preferred directions.

First, we needed to define a reference to the closest boundary, relative to which we could measure the orientation of the branches. For this purpose, we computed the gradient field of the distance transform on the intraspine volume (excluding organelles). As the distance transform is smallest at the cell membrane and reaches its maximum at the center, the gradient vectors point away from the membrane toward the center of the volume. We computed the relative orientation of all filaments with respect to the gradient vector field (Fig. 6*A*). One orientation-value was computed for each branch (a filament connecting two nodes).

Heads and necks of all spines show similar distributions of the relative branch orientation (Fig. 6*B*). The average branch orientation is slightly lower in hippocampal spines (mean ± STD: 59.0 ± 1.7°). Cerebellar spine heads and necks had mean orientations of 60.9 ± 0.8° and 61.5 ± 1.2°, respectively. In necks of hippocampal pyramidal cells, we found a mean value of the average orientation of 61.4 ± 2.3°. The mean relative orientation was slightly but significantly higher in necks (61.5°) than in spine heads (59.3°; *p* = 0.002). Mean values ranged from 57.5° in the head of spine H2 up to 70.1° in the particularly narrow neck of spine H11.

The angle distribution for randomly oriented filaments scales as sin(α), so that the mean relative orientation is 57.3° (Fig. 6*D*, blue line). Filaments running parallel to the main axis of the spine neck would shift the angle distribution toward 90°. Combining all branch orientations for all spines, a small shift toward values close to 90° was observed, although the random-orientation model was still a fairly good description (Fig. 6*D*).

To understand how deviations from the random-orientation model could arise, we approximate all branches as straight, rigid rods of a fixed length of 15.4 nm (Fig. 6*E*, blue line, *A*, inset), which corresponded to the average branch length (Fig. 3*B*,*C*). As orientation angles are measured relative to the center of each rigid rod, if this center is closer than 7.7 nm to the membrane, the possible orientations are restricted; in fact, if the center is directly next the membrane, the rod must lie parallel to the membrane, forming a 90° angle with the gradient of the distance transform. In light of this rigid-rod model, we examined the mean branch orientation with respect to the distance to the closest membrane (Fig. 6*E*). As predicted, close to the membrane the average relative orientation increased, indicative of a preference for filaments to align with the boundary. Further away from the boundary, the orientations were no different from random.

A close inspection of the tomograms revealed the existence of putative actin filaments that maintain a common orientation across multiple nodes. In thin spine necks (compare H1, H10, and H11 in Fig. 2*A*), such straight filaments are occasionally discernible (Fig. 6*H*) and tend to align with the neck’s main axis. In contrast, little evidence of alignment was found in stubbier spines with either wider or shorter necks. Examples of this latter phenomenon are shown in Figure 6*F*,*G* for spines C3 and H9. Despite this particular effect of spine neck width on orientation, other measures such as branch lengths, node distributions and branching angles did not have different statistics as a function of neck width.

In summary, for very thin necks whose diameter is close to the average branch length, the filament mesh within the neck can only adopt a restricted set of configurations, notably in regard to branch orientation. Otherwise, branches had no preferred orientation.

### Elementary loops

The postsynaptic cytoskeleton not only maintains a spine’s shape, but its filigree structure also likely affects what molecules can pass through the mesh. To understand the size of “holes” in the cytoskeleton *in situ*, we analyzed loops of filaments (Fig. 7*A*). Loops are defined as closed paths in the graph composed of the branch nodes as vertices and the branches as edges. On average, we found between eight and nine nodes or branches in an elementary loop (Fig. 7*B*).

The loop lengths are broadly distributed (Fig. 7*C*), just like the branch-lengths themselves are variable (Fig. 3*C*). The mean loop length of the pooled distribution across all spines was 122 nm with a STD of 38 nm. The mean loop length of the individual spine domains (Fig. 7*C*, red lines) varied between 107 and 141 nm. There was no difference between heads and necks (pyramidal cells: *p* = 0.63, Purkinje cells: *p* = 0.96). Moreover, although the mean loop-length was longer in Purkinje cells than in pyramidal cells in both heads (128 ± 7.3 vs 120 ± 7.2 nm, mean ± STD, *n* = 5, *n* = 11) and necks (128 ± 5.7 vs 122 ± 8.0 nm, mean ± STD, *n* = 5, *n* = 11), this difference was not statistically significant (*p* = 0.08, *p* = 0.10).

The plane that best fits a given loop (Fig. 7*A*) is given by the first two principal components of the node positions along that loop. As shown in Figure 7*D*, the first two principal components account on average for 95% of the variance in the node positions. This means that the loops deviate only slightly from planarity (see also Fig. 7*E*). These two principal components were not identical in magnitude, indicating that typical loops are not circularly symmetric; instead, the ratio of the primary to the secondary axis is ε = 1.47 ± 0.40 (mean ± STD).

### Spanning trees

**Figure 8.**
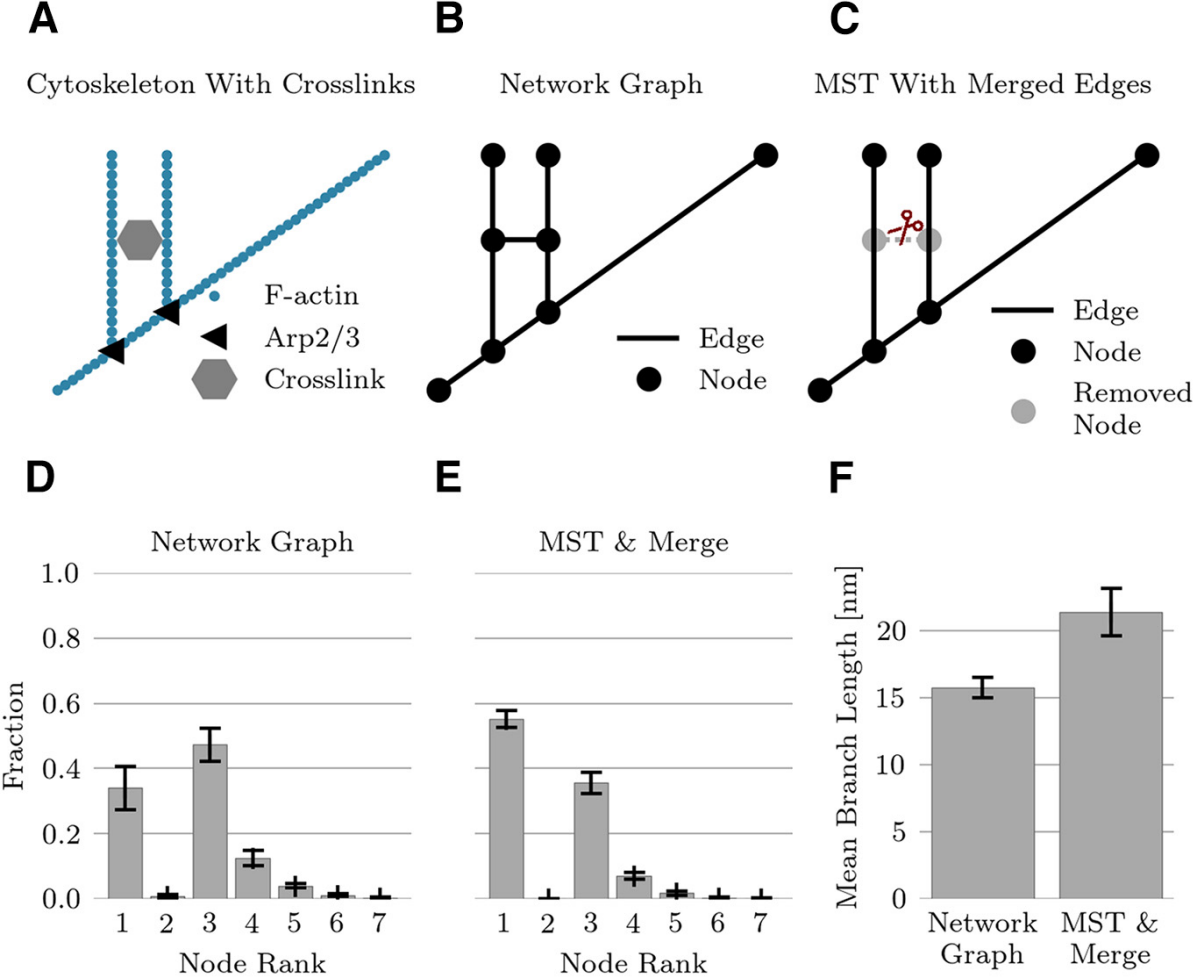
Pruned network. ***A***, Actin cytoskeleton is cross-linked by various proteins. ***B***, Cross-links create additional nodes and edges in the graph representation of the network. ***C***, Computation of a minimum spanning tree (MST) with subsequent joining of edges connected by a rank-2 node leads to a tree-like structure without any loops. ***D***, The network graph has 34% rank-1 nodes (branch ends). ***E***, The pruned graph structure contains no loops and has 55% rank-1 nodes. ***F***, The mean branch length increases from 15.7 nm in the network graph to 21.3 nm in the pruned graph (sample size *n* = 31, errorbars indicate STD in panels ***D–F***).

Cross-links were presumably responsible for creating loops in the reconstructed cytoskeleton, which would not be present in the purely branching structure of actin induced by Arp2/3 and capping proteins (Fig. 8*A*). Even in the absence of specific markers for cytoskeletal proteins, one can recover a branching structure from the reconstructed graph. Under the hypothesis that cross-linking filaments are shorter than typical actin filaments, we preferentially removed short branches by minimizing the sum of the inverse branch lengths (Fig. 8*B*). The construction of such a minimum spanning tree removes all loops in the network meshes. Moreover, in any tree graph, the number of nodes is equal to the number of branches plus one. In a second step, edges connecting to a rank-2 node (which emerged after pruning) were joined together into a single edge (Fig. 8*C*).

Averaged across all spine domains in the data set (*N *=* *31), the minimum spanning trees occupied 17.5 ± 2.2% (mean +/– STD) of the total volume, compared with the 24.5 ± 3.7% (mean +/– STD) occupied by the original graphs. By eliminating branches and then any resulting rank-2 nodes, the branch density was reduced by 41 ± 8% (mean +/– STD). In the minimum spanning tree, the node density is effectively the same as the branch density. Eliminating rank-2 nodes corresponded to a reduction by 24 ± 6% (mean +/– STD) in the number of nodes. Merging the edges attached to the rank-2 nodes of the minimum spanning tree increased the mean branch length from 15.7 ± 0.9 to 21.4 ± 1.8 nm (mean +/– STD) (Fig. 8*F*). In both the original reconstructed networks and the minimum spanning trees, the branch ends (rank-1 nodes) and bifurcation points (rank-3 nodes) dominated (Fig. 8*D*,*E*).

## Discussion

This first quantitative analysis of the 3D fine structure of dendritic spines based on *in situ* specimens was possible thanks to advances in specimen preparation methods, such as high-pressure freezing, and advances in imaging provided by high-resolution multitilt serial section electron microscopic tomography. Unlike platinum-replica-based imaging by EM, as used, for instance, by Korobova and Svitkina (2010), our approach did not require solubilization of the plasma membrane to expose the cytoskeleton and partial extraction of cytoplasm before metal shadowing. Methods developed for this study allowed us to analyze preparations derived from *in situ* brain tissue without requiring the use of the simplified and incompletely differentiated synaptic complexes typical of cell culture and work being done using cryoEM. Glutaraldehyde fixation before high-pressure fixation and freeze substitution minimized the extraction of soluble cytoplasmic proteins, which meant that these were also stained. The ultrastructure we extracted was, therefore, not exclusively composed of actin and actin-binding proteins. A quantitative study comparing cryoEM to aldehyde fixation finds that hippocampal spine neck diameters are 50% larger after aldehyde fixation (Tamada et al., 2020), which indicates a possible role of spurious cross-links arising from cytoplasmic proteins in expanding the actin network. In our hands, however, the measured spine neck diameters were comparable to the cryoEM measurements of Tamada and colleagues. Moreover, prior studies have demonstrated the success of our approach in identifying actin (Sosinsky et al., 2008; Burette et al., 2012). Using STED microscopy, spine necks in cultured neurons exhibit regions of high actin density alternating with regions of high spectrin density (D’Este et al., 2015; Bär et al., 2016); as our EM staining did not specifically target these proteins, we could not confirm this finding in *ex vivo* samples.

Using a combination of image binarization and topological thinning, we tracked filaments traversing both the actin branches and numerous cross-links. The reconstructed mesh, therefore, extended beyond F-actin and its branches, with the cross-linking proteins creating a uniform actin-polymer gel throughout all spine subdomains. Despite differences in size between cerebellar and hippocampal spines, the statistical properties of the cytoskeletal ultrastructure were quite similar in both cell types.

Except in the direct vicinity of the membrane, where mechanical stresses deform the cytoskeleton, the orientation of the presumed actin branches was completely random (Fig. 6*B*,*C*). We found little evidence for unbranched parallel-running fibers (Lei et al., 2016) in the spine neck. The distance between branch points was only 15.4 nm on average (Fig. 3*B*,*C*), and branches were considerably bent, as measured by their tortuosity (Fig. 3*D*,*F*).

Most nodes (72%) linked three branches, indicating that the predominant feature of the filamentous architecture is bifurcation (Fig. 4*B*). The Arp2/3 complex *in vitro* nucleates new actin filaments that branch off at 70° from the main filament, but the branching patterns at rank-3 nodes in the reconstructed network differ: instead of a planar 70°−110°−180° configuration one finds *in vitro*, many branchings at rank-3 nodes no longer lay in a single plane, and while the intermediate angle frequently was close to 110°, the other angles bent away from the *in vitro* angles (Fig. 5). Although some of the angles made at rank-3 nodes are likely made by cross-linking proteins such as filamin A, CaMKII, or nonmuscular myosin IIb (van der Flier and Sonnenberg, 2001; Rex, Gavin et al., 2010; Khan, Downing et al., 2019), or be unrelated to actin, one plausible hypothesis is that mechanical forces from membrane surfaces deform the actin mesh. A previous study finds that the nucleation of new actin branches is sensitive to mechanical cues, with daughter branches preferentially attaching to the opposite side of the direction of bending (Risca et al., 2012), a finding that is consistent with our data.

We could recover a purely branching structure for the network mesh by computing a minimum spanning tree, which removed loops in the network mesh. As some cross-linking proteins such as filamin or fimbrin are shorter and narrower filaments (Hanein et al., 1998; Nakamura et al., 2007) than actin, so that these are treated as “decorating” actin, the objective function for the minimum spanning tree was designed to preferentially remove short branches. Edges connecting to a rank-2 node (which emerged after pruning) were joined together into a single edge and the rank-2 node was removed from the graph. The node density dropped by nearly one-quarter after pruning. As a direct consequence of pruning, the branch and node densities were the same. The true node density will determine the mechanical properties of the cytoskeleton: as reviewed in Hohmann and Dehghani (2019), the elastic modulus scales inversely with the number of nodes raised to the fourth power. Branching angle distributions remained qualitatively the same in the original graph and the minimum spanning trees; and the node density remained constant throughout space in spine necks and heads, indicative of a spatially uniform gel.

Most differences in statistical measures of the internal structure between spine necks and heads could be explained by differences in the surface-to-volume ratio, with the exception of a higher density of putative actin and nodes in spine heads (Figs. 2*E*, 4*D*). These differences were minor, however, and smaller in magnitude than the STD across samples. Taken together, these results provide evidence against the traditional view that spine heads and spine necks possess distinct actin architectures (Halpain, 2000; Tada and Sheng, 2006; Hotulainen et al., 2009). The *in vitro* studies of Korobova and Svitkina (2010) have already shown that the actin-cytoskeleton undergoes a more gradual and less drastic change in its architecture from the spine head to the neck. Our *in situ* data go a step further and demonstrate that the cytoskeleton is a uniform gel down to the nanometer scale.

The cytoskeleton in spines must serve two (possibly) opposing purposes: confer mechanical stability and permit the passage of molecules through the gel. The mechanical properties of an actin mesh together with its associated binding proteins, as opposed to single actin filaments, are not at all understood. Filaments are rigid to thermal fluctuations on length scales smaller than 10 μm (McCullough et al., 2008). The flexural rigidity of F-actin is estimated to be κ = 0.040 pN μm^2^. From mechanics, the compressive force *F* needed to buckle a filament of length *L* is at least *F* = π*^2^* κ/*L^2^*. For instance, at least 0.4 pN are needed to buckle a 1-μm-long filament (see also Blanchoin et al., 2014, their Fig. 2C). Forces required to rupture crosslinks can be as high as 30 pN for α-actinin and 50 pN for filamin (Blanchoin et al., 2014). Actin-binding proteins can affect actin’s rigidity; cofilin, for example, decreases the rigidity by a factor of ∼5 (McCullough et al., 2008). Extrapolating the values for F-actin to the mesh is fraught with difficulty, however.

Both the spine geometry and the specific organization of the cytoskeleton likely influence the diffusion of molecules through the spine (Tønnesen et al., 2014). Indeed, synaptopodin changes the cytoskeleton and increases the diffusion time constant of metabotropic mGluR5 receptors (L Wang et al., 2016). If one treats the actin gel as a rigid structure, it poses a barrier to the movement of larger molecules.

In a back-of-the-envelope calculation, one computes the size of typical “pores” in the actin structure by dividing the typical loop length of ∼120 nm by π·ε (ε = 1.47 is the average elongation factor for the best-fit ellipse to the loop) and then subtracts the actin width (∼8 nm). The resulting short-axis diameter is ∼18 nm. A CaMKII molecule in its compact (nonactivatable) form could pass through such a pore, as CaMKII in this form has a gear-shaped central body with a height of ∼10 nm and a diameter of ∼14 nm (Kolodziej et al., 2000). In contrast, CaMKII in its activation-competent conformation ranges from 15 to 35 nm in diameter (Myers et al., 2017); the cytoskeleton could hinder the passive diffusion of CaMKII in this state.

But the cytoskeleton is likely more than a simple physical barrier to diffusion. The uniform structure described in the present study could be the starting point for theoretical studies of electrodiffusion to understand the interaction between actin’s dense negatively charged domains and motile polar and charged molecules, including water, using multiscale methods (Ververis and Schmuck, 2017). Transient changes in the electric field across the spine, associated with synaptic activation, lead to rapid remodeling of actin and spine geometry (Bloodgood and Sabatini, 2005; Hlushchenko et al., 2016), during which the transmembrane protein CD44, the link between the actin cytoskeleton and the extracellular matrix (Tsukita et al., 1994; Nagano and Saya, 2004; Stawarski, et al., 2014; Roszkowska et al., 2016), is likely cleaved by matrix metalloproteinases, secreted following synaptic activation. Therefore, the gel structure of actin *in vivo* could play a role not only in creating electrical and chemical microdomains in spines but also affect synaptic plasticity and even long-term memory (Tsien, 2013).

## Synthesis

Reviewing Editor: Alexey Semyanov, Shemyakin-Ovchinnikov Institute of Bioorganic Chemistry Decisions are customarily a result of the Reviewing Editor and the peer reviewers coming together and discussing their recommendations until a consensus is reached. When revisions are invited, a fact-based synthesis statement explaining their decision and outlining what is needed to prepare a revision will be listed below. The following reviewer(s) agreed to reveal their identity: NONE. Note: If this manuscript was transferred from JNeurosci and a decision was made to accept the manuscript without peer review, a brief statement to this effect will instead be what is listed below.

I have assessed the reviewers’ comments in J Neuroscience and believe that the paper can be accepted for publication in eNeuro.
