# Supplementary figures and images for: A Uniform and Isotropic Cytoskeletal Tiling Fills Dendritic Spines
Source: eNeuro. 2022 Oct 26;9(5):ENEURO.0342-22.2022. doi: 10.1523/ENEURO.0342-22.2022 (PMC9617608; doi:10.1523/ENEURO.0342-22.2022)

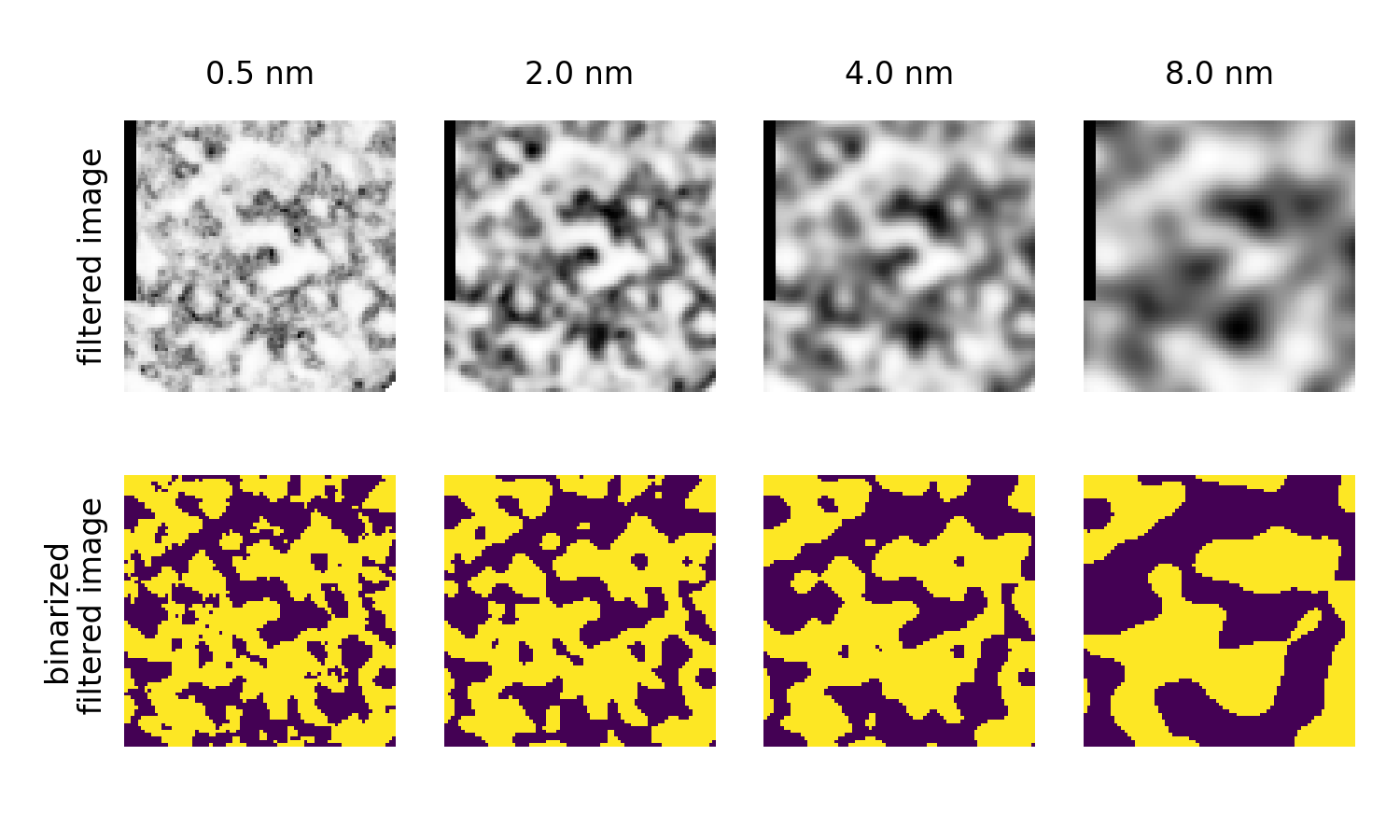

Supplement: Extended Data Figure 1-1 — Effect of filter width. Image noise was reduced by smoothing the tomogram with a Gaussian filter. The best results were obtained with a SD of 2 nm for the filter. Smaller SD s are not sufficient to eliminate noise and larger SD s do not preserve the topology of the cytoskeleton. Scale bar: 100 nm. Download Figure 1-1, TIF file. [file enu-eN-NWR-0342-22-s01.tif]

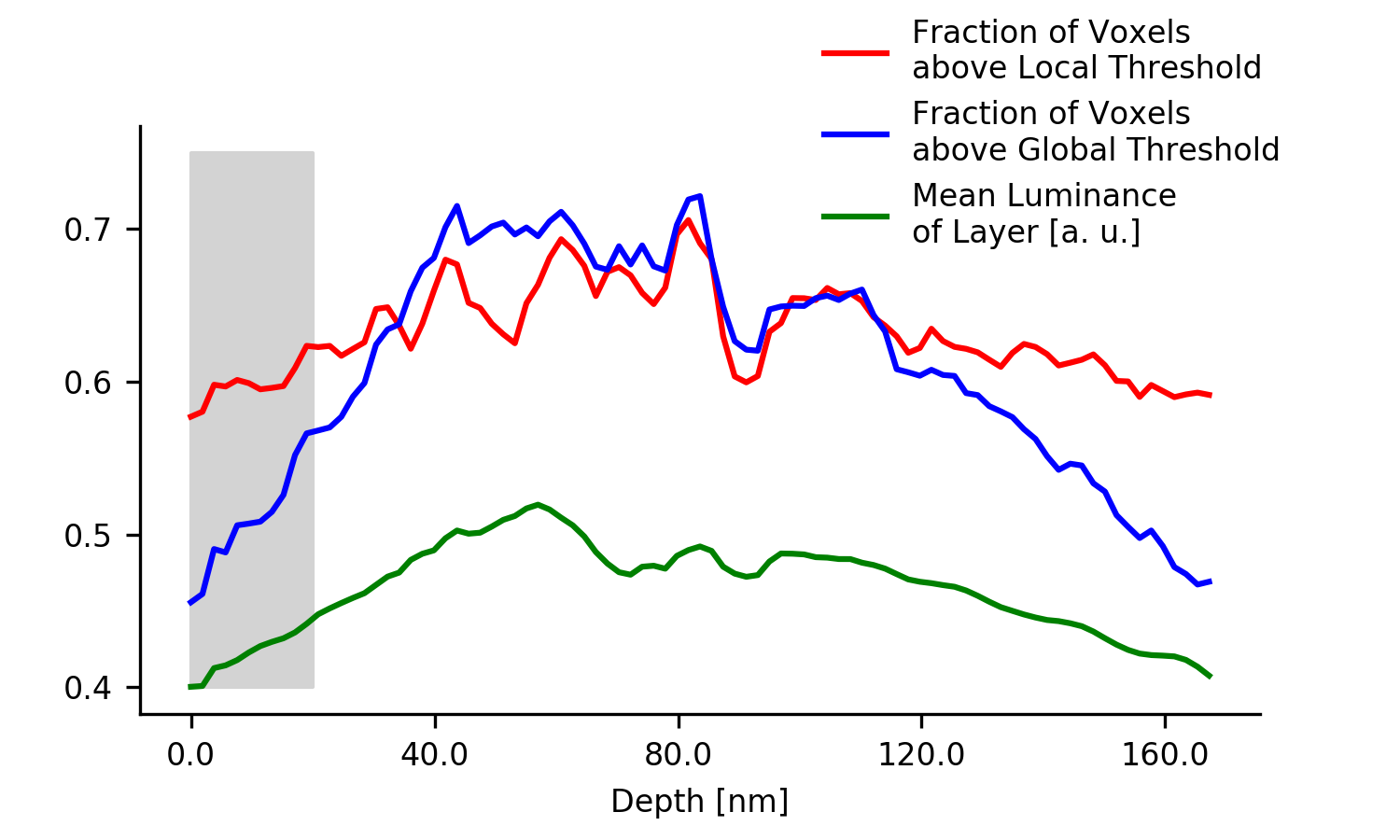

Supplement: Extended Data Figure 1-2 — Local threshold binarization. A threshold is used to binarize the image into strongly and weakly stained voxels (see Extended Data Fig. 1-1). The mean luminance of a virtual slice through the tomogram varies along the z-axis. As the local luminance changes, the number of voxels above a fixed threshold changes, so we used a local threshold. The width of the gray area represents the size of the (sliding) window used for the computation of the local threshold. Download Figure 1-2, TIF file. [file enu-eN-NWR-0342-22-s02.tif]

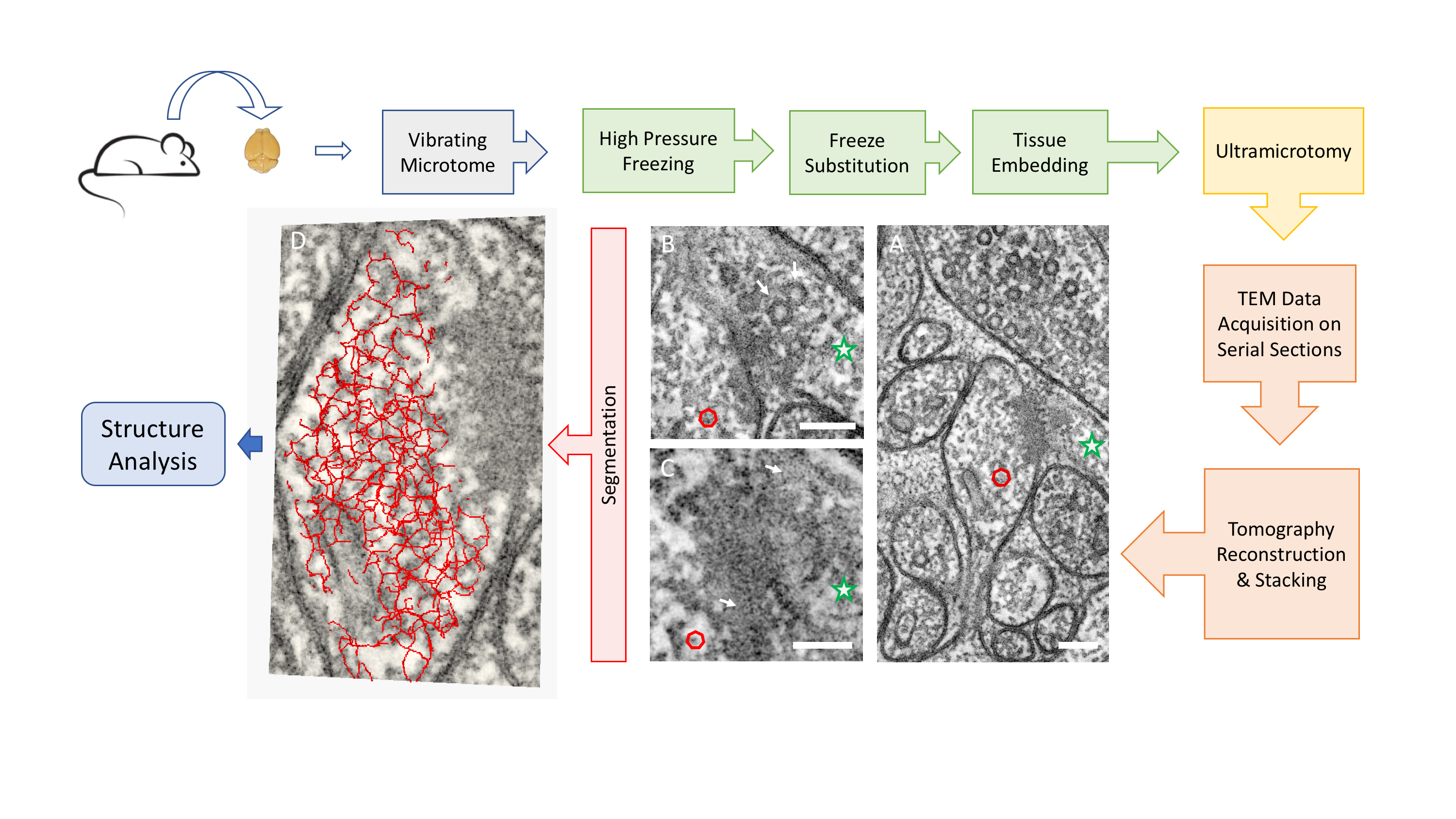

Supplement: Extended Data Figure 1-3 — Coarse outline of the workflow. Following perfusion, a mouse brain is sectioned into thick (100 μm) slices using a vibrotome. Portions of interest are then punched out from brain slices (C stands for cerebellum, and H for hippocampus), before being processed through high pressure freezing and freeze substitution. This experimental protocol allows for an optimal sample preservation, which when combined with a multiple tilt EM tomography scheme on thin slices will provide high-quality reconstruction of the brain. Images A–C represent snapshots of the synapse corresponding to spine H11 taken at three different depths. The green stars (respectively, red circles) point to the presynaptic (respectively, postsynaptic) terminal. Note the overall quality of the synaptic vesicles (white arrow on panel B) as well as the quality of the membrane definition (white arrow on panel C) in this en face view. Once segmented out, the spine (red circles) is skeletonized and the resulting mesh, characteristic of the internal structure of the spine, is subsequently analyzed with the methods outlined and developed in this paper. Download Figure 1-3, TIF file. [file enu-eN-NWR-0342-22-s03.tif]

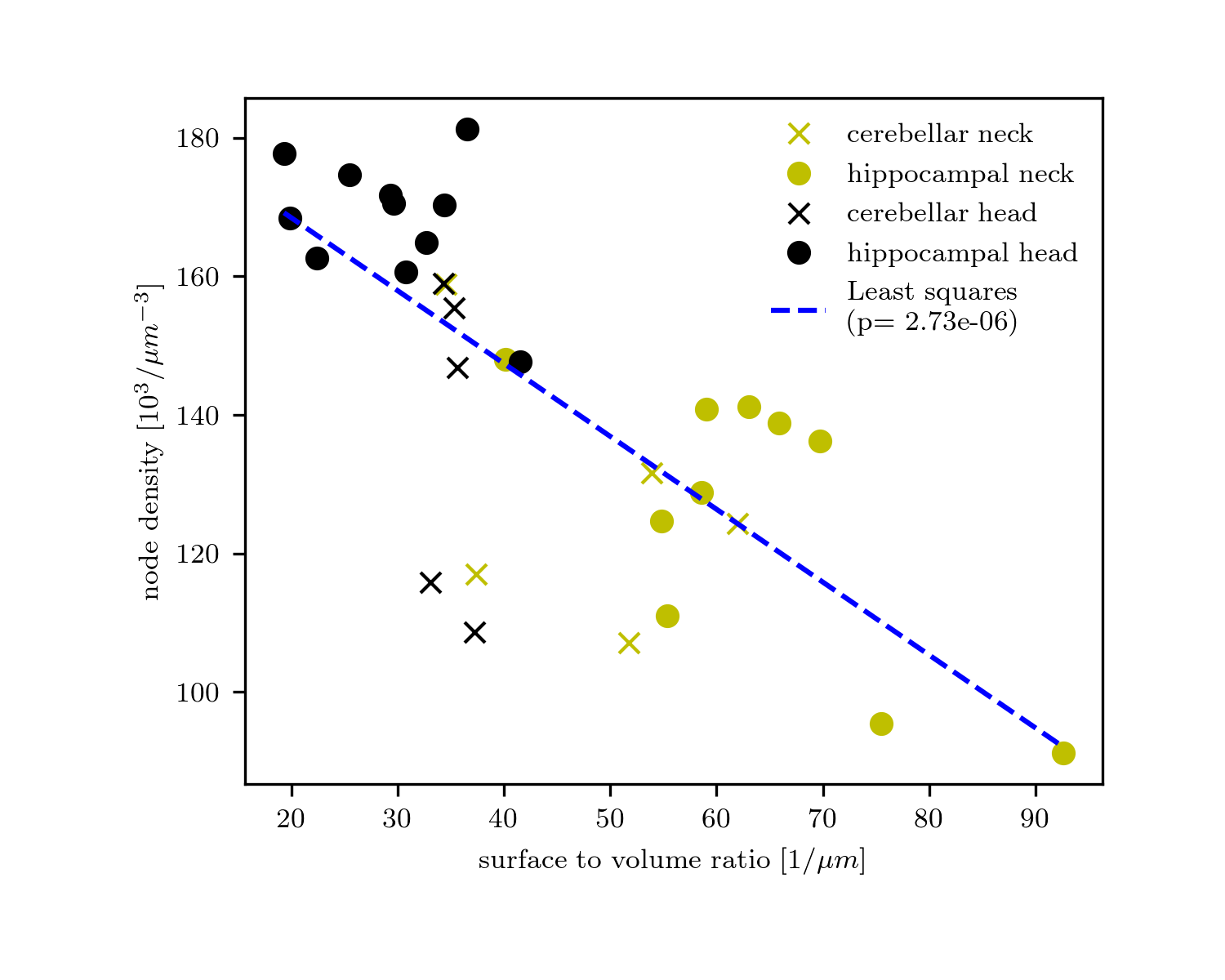

Supplement: Extended Data Figure 4-1 — Correlation between surface-to-volume ratio and node densities. The surface-to-volume ratio is a good predictor of the average node density within a spine. Download Figure 4-1, TIF file. [file enu-eN-NWR-0342-22-s04.tif]
